# How Frost Forms and Grows on Lubricated Micro- and Nanostructured Surfaces

**DOI:** 10.1021/acsnano.0c09152

**Published:** 2021-03-01

**Authors:** Lukas Hauer, William S. Y. Wong, Valentina Donadei, Katharina I. Hegner, Lou Kondic, Doris Vollmer

**Affiliations:** † Physics at Interfaces, Max Planck Institute for Polymer Research, Ackermannweg 10, 55128 Mainz, Germany; ‡ Faculty of Engineering and Natural Sciences, Tampere University, P.O. Box 589, FI-33014 Tampere, Finland; § Department of Mathematical Sciences and Center for Applied Mathematics and Statistics, New Jersey Institute of Technology, Newark, New Jersey 07102, United States

**Keywords:** wicking, icing, condensation frosting, thin film, frost percolation, slippery surface slips

## Abstract

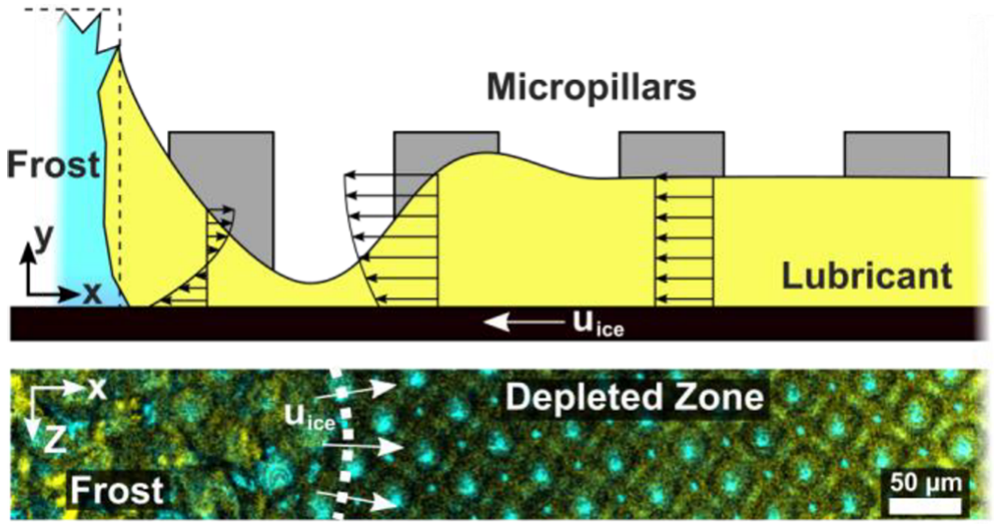

Frost is ubiquitously observed in nature whenever warmer and more humid air encounters colder than melting point surfaces (*e*.*g*., morning dew frosting). However, frost formation is problematic as it damages infrastructure, roads, crops, and the efficient operation of industrial equipment (*i*.*e*., heat exchangers, cooling fins). While lubricant-infused surfaces offer promising antifrosting properties, underlying mechanisms of frost formation and its consequential effect on frost-to-surface dynamics remain elusive. Here, we monitor the dynamics of condensation frosting on micro- and hierarchically structured surfaces (the latter combines micro- with nano- features) infused with lubricant, temporally and spatially resolved using laser scanning confocal microscopy. The growth dynamics of water droplets differs for micro- and hierarchically structured surfaces, by hindered drop coalescence on the hierarchical ones. However, the growth and propagation of frost dendrites follow the same scaling on both surface types. Frost propagation is accompanied by a reorganization of the lubricant thin film. We numerically quantify the experimentally observed flow profile using an asymptotic long-wave model. Our results reveal that lubricant reorganization is governed by two distinct driving mechanisms, namely: (1) frost propagation speed and (2) frost dendrite morphology. These in-depth insights into the coupling between lubricant flow and frost formation/propagation enable an improved control over frosting by adjusting the design and features of the surface.

Water is widely available in the atmosphere and in the environment. The interplay between the chemical properties of water and the thermal conditions of our planet results in the formation of clouds, rain, or frost.^1,2^ Despite the ubiquitous nature of frost, understanding and controlling its formation^3^ and propagation^4,5^ still poses several challenges. Control of frosting is relevant for a range of industries: In the energy,^6^ transportation,^7^ or telecommunication^8^ sectors, frost constitutes serious hazards when it forms on critical components of machines and devices, causing them to fail. To control frosting, a strong momentum of research in anti-icing technology^9−12^ was recently generated. Surfaces which passively avoid, repel, or retard frost or ice formation were designed.^13−16^ Lubricant-infused surfaces (LIS) were shown to resist frost formation more efficiently compared to dry micro/nanostructured or flat, chemically functionalized variants.^16^ Lubricant-infused porous surfaces are characterized by solid three-dimensional (3D) structures, infused with a liquid lubricant.^17,18^ The lubricant renders the surface slippery, resulting in a high mobility of contacting liquids. Capillary forces retain lubricants within the surface by virtue of the porous, structured geometry.^19^ During condensation frosting, however, the lubricant in the porous structure of LIS appears to reorganize, leading to depletion of the structure.^20,21^ Aiming to gain insight in the underlying dynamics, here, we monitor and model lubricant reorganization and frost propagation dynamics (space- and time-resolved) on micro- and hierarchically (combining micro- with nanofeatures) structured surfaces under moderately low subzero temperatures (−12° to −22 °C).

Despite the widespread occurrence of frost, the understanding of its formation and growth—particularly on LIS—remains elusive. The reasons are multifold: Insights into frosting are hampered by poor optical contrast between frost and lubricant and by the nonequilibrium nature of frost formation that involves multiple time and length scales. Mathematical modeling of the involved nonequilibrium processes requires the description of freezing, dendrite growth, propagation of frost bridges, and lubricant flow in mutual interplay. The first clue of lubricant reorganization was obtained by focused ion beam and scanning electron microscopy, revealing that frozen water droplets on LIS are covered by a thin layer of lubricant.^20^ However, none of these techniques and investigations allow for insights into the coupled evolution of both frost and lubricant.

To reach better insight, we have developed a setup that enables the *in situ* monitoring of lubricant reorganization and frost formation using laser scanning confocal microscopy (LSCM). Such a setup allows to discriminate between frost and lubricant. In particular, our setup provides the spatially and temporally resolved data of the formation and growth of frost, accompanied by quantifiable lubricant reorganization. The summary of our findings is that analogously to frost propagation on dry surfaces, frost bridges^4,22^ form. A strong capillary pressure inside the frost structure induces lubricant flow during condensation frosting. Three-dimensional surface mapping by quantitative confocal microscopy provides a time-resolved evolution of lubricant during frosting. We then utilize a long-wave approximation^23^ to model the experimentally quantified lubricant reorganization during the transient process of frost formation and propagation. We derive and solve the governing equations numerically to predict the height profile of the lubricant. The experimental data and numerical predictions for lubricant reorganization on micro- and hierarchically structured surfaces show excellent agreement. Notably, we find that lubricant reorganization and depletion is primarily affected by the frost propagation speed and dendritic frost geometry.

## Results and Discussion

**Figure 1:**
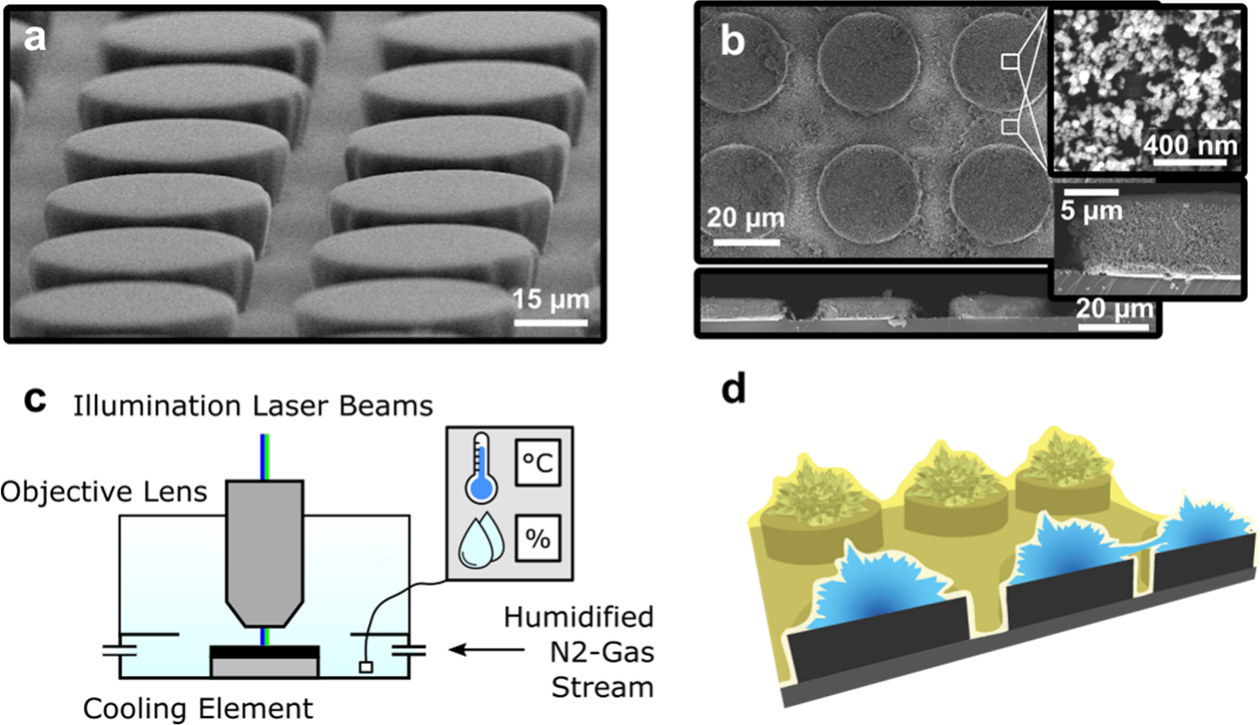
Condensation frosting on lubricated micro- and hierarchically structured surfaces. (a) Scanning electron microscope (SEM) image of the plain micropillar surface. The pillars have a diameter of 30 μm, a height of 10 μm, with a separation distance of 10 μm. (b) SEM images of hierarchical structure consisting of the micropillar array coated with silica nanoparticles on the entire surface. Highly magnified image (top right) of the particle coating shows that individual particles have diameters of <50 nm. The entire surface is evenly coated with nanoparticles, while the microstructure is retained (bottom). (c) Experimental setup: The surface is mounted on the cooling element and monitored through an objective lens (2.5×, 10×, 100×). A green (532 nm) and a blue (473 nm) laser are used to illuminate the sample. A humidified carrier gas (nitrogen) is introduced into the chamber. During the experiment, the temperature and humidity in the far field are recorded. (d) Schematics of condensation frosting (blue) on the infused (yellow) micropillar substrate.

Condensation frosting on lubricated micro- and hierarchically structured surfaces is temporally and spatially resolved by LSCM. As model surfaces (approximately 5 cm^2^ large), we use regularly spaced micropillar arrays (square orientation) (Figure 1). To analyze the influence of nanoroughness on condensation frosting, the lubricant distribution is monitored on bare micro (Figure 1a) and hierarchically structured surfaces, characterized by both micro and nanoscopic features. Here, the hierarchical structure is facilitated by coating the micropillar arrays with a thin layer of silica nanoparticles (Figure 1b). The cylindrical micropillars are fabricated by photolithography using an epoxy-based photoresist (SU-8, *cf*. Methods). Two variants of functionalized silica nanoparticles (amine and epoxy groups) are then deposited in a “layer-by-layer” method, *via* a two-step dip-coating procedure (*cf*. Methods). This creates a thin, homogeneous nanoparticle coating of approximately 1 μm thickness on the micropillar array. The average roughness on top of the micropillars increased from 3.4 ± 0.3 nm to 35.1 ± 1.7 nm, after the nanoparticle coat was added (Figure S1). We then infuse the surfaces with 2 μL of fluorescence marked silicone oil (viscosity, *η*_SiOil_ = 194 mPa s) as a lubricant. After leaving the surface overnight, homogeneous lubricant distribution in the pillars’ interstices (approximately 10 μm height) with a thin layer on top of each pillar (below 1 μm) is verified by confocal microscopy. The LIS is placed on a cooling element in a sealed (frosting) cell and directly monitored simultaneously using a custom-built confocal microscope (Figure 1c, Figure S2, Methods). We cool down the surfaces to set point temperatures below the atmospheric freezing point of water (Figure S3) in a dry atmosphere (<4% relative humidity, RH) which were then held constant for 10 min. To trigger condensation frosting (Figure 1d), we flow a humidified nitrogen carrier gas (30% RH at 18.1 ± 0.6 °C, Figure S4, Methods) for 30 s into the cell. Thereafter, the cell is sealed. The long working distance of all objective lenses (100×/0.80:2 mm; 10×/0.4:3.1 mm, 2.5×/0.07:9 mm) provides sufficient space between lens and sample to ensure evenly distributed humidity around the sample substrate. The laser illumination is non-invasive and does not interfere with the observed processes on the sample substrate (*cf*. SI S1).

### Condensation on Microstructured LIS

**Figure 2:**
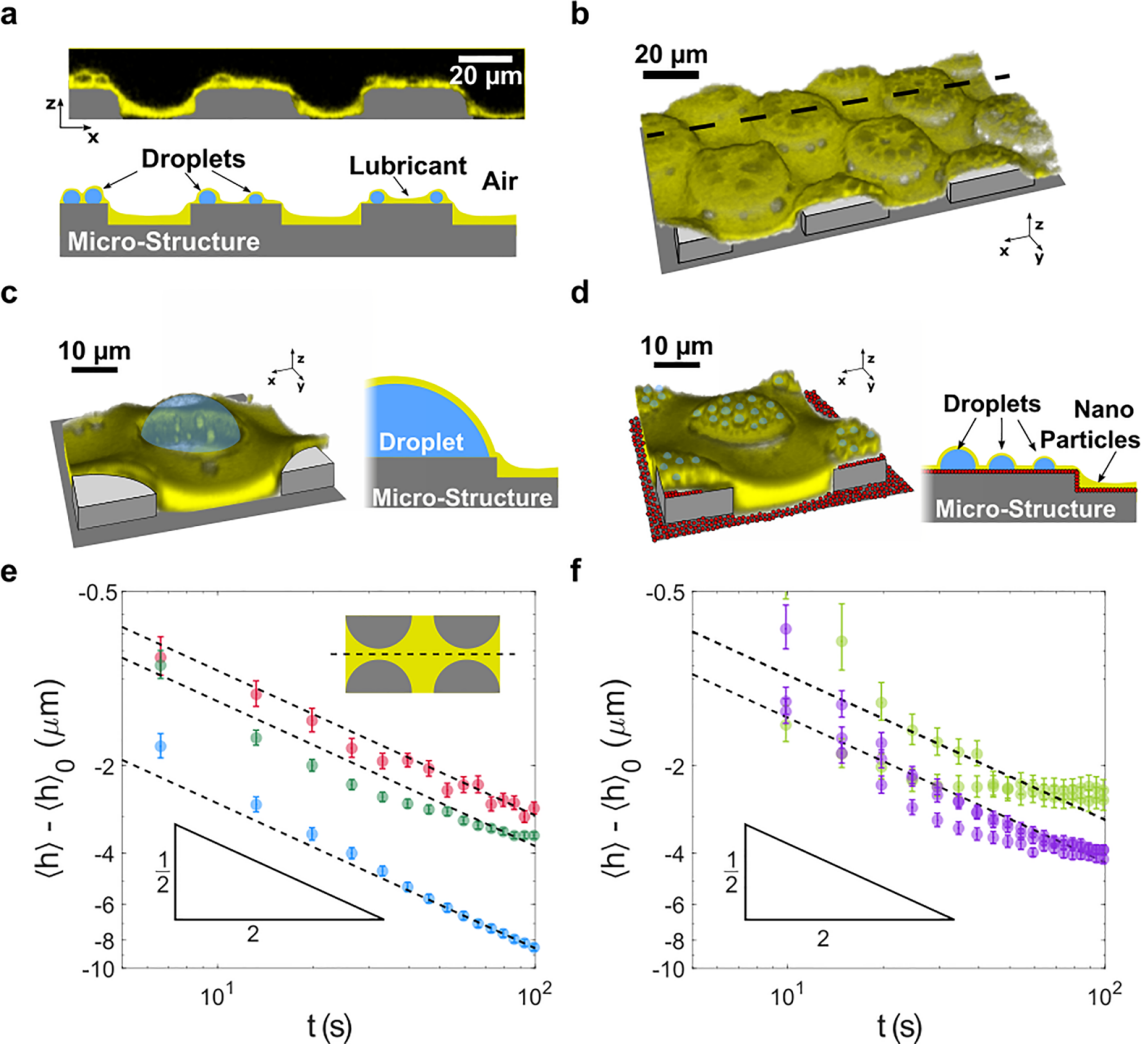
Condensation of water droplets on lubricant-infused, micro- and hierarchically structured surfaces. The silicone oil (yellow) is dyed with the fluorophore. Condensed water, the surfaces’ micro- and nanostructures, and the surrounding air do not fluoresce and appear dark. (a and b) Condensing droplets on bare microstructured surfaces. The droplets become initially visible as spheroids on the pillars’ tops, outlined by the fluorescent lubricant with LSCM. (a) Top: Vertical cross section through micropillars. Several droplets form on each pillar’s top face. Bottom: Illustration of the initial condensation recording for clarification. (b) 3D view of the surface, showing lubricant distribution and condensed water droplets on the pillars’ top (dark spots). (c) On bare microstructured surfaces, condensing droplets coalesce, until a single, lubricant covered droplet resides on each pillar top, covered with lubricant. The yellow spots on the lubricant coat are either caused by oil patches or dye aggregates. (d) On hierarchically structured surfaces, multiple drops remain on the pillars top faces. Illustrations of respective surfaces and condensate droplets (*cf*. Figure S5 for raw data for (c and d)). (e) Averaged lubricant displacement ⟨h⟩ – ⟨h⟩_0_ over time for (e) micro- and (f) hierarchically structured surfaces, within the first 100 s of the experiment. ⟨h⟩_0_ = 10 μm refers to the lubricant height at *t* = 0 s. The height of the lubricant in between the pillars ⟨h⟩(*t*) is spatially averaged over the dashed line (inset). Substrates are cooled down to −12 °C (red), −14 °C (dark green), 17 °C (light green), −20 °C (blue), and −22 °C (purple).

Approximately 10 s after introducing the stream of humidified nitrogen into the frosting chamber, the formation of distinct and individual droplets on micropillar tops becomes visible (dark spheroids) (Figure 2a,b). Vertical cross-sectioning through the surface shows condensed droplets wrapped in lubricant and the accompanied depletion of lubricant between the micropillars (Figure 2a). A 3D image reveals that the droplets (dark spots) are formed on top of the micropillars (Figure 2b). The thin lubricant film on top of the micropillars is permeated easily by atmospheric water vapor molecules *via* diffusion (*D*_W/SiOil_ = 2 × 10^–9^ m^2^/s) and accumulates at the micropillar–lubricant interface due to the finite solubility of water in silicone oil of up to 40 mM.^24^ Here, stable nucleation sites for condensation are most likely due to sufficiently low energy sinks at this location (*cf*. SI S2).^25−28^ After nucleation, the droplets grow in size and soon become visible under the microscope: Droplets appear as dark spheroids, surrounded by lubricant (yellow) (Figure 2a,b). At the contact line where the droplet, lubricant, and air meet, the surface stresses of the respective phases fulfill a force balance.^29^ The vertical component of the droplet surface stress promotes the formation of annular wetting ridges^30^ of silicone oil around condensing water droplets. Due to a positive spreading parameter,^31^*S* = *γ*_*sv*_ – (*γ*_*sl*_ + *γ*_*lv*_) ≈ 12 mN/m^15^ silicone oil spreads on water, leading to completely cloaked^32,33^ droplet–air interfaces by lubricant. The lubricant layer around each droplet (ridge and cloak) slightly retards coalescence.^34^ However, when the droplets grow, the layer thins until it becomes unstable and disintegrates, leading to coalescence. Eventually, only one single droplet (diameter ≈30 μm) remains on top of each micropillar (Figure 2c).

### Condensation on Hierarchical LIS

While the initial emergence of femtoliter droplets was similar on both surface types, the consecutive droplets growth phase deviates on hierarchically structured surfaces. The separating lubricant layer around droplets appears to be more stable. On hierarchically structured surfaces, not a single droplet but multiple sessile droplets remain on top of each pillar (Figure 2d). The increased surface roughness introduces pinning sites for the droplets, leading to delayed coalescence. Hence, the nanoparticle coat prevents the coalescence of condensate droplets into a single one.

To quantify the migration of lubricant during condensation, we monitor the film height in the array’s interstices, that is, in a square domain of 40 μm × 40 μm, containing 4 half micropillars (field of observation) (Figure 2e inset). The lubricant height *h* is evaluated at *n* = 128 spots along the observed length (inset, dashed line), while the temporal resolution is 0.2 frames per second. The time dependence of the average height,, is shown for micro- (Figure 2e) and hierarchically structured (Figure 2f) surfaces, respectively. For microstructured surfaces, the average lubricant height depletes according to a square root behavior, ⟨*h*⟩ ∼ *t*^–1/2^ (Figure 2e), from the initial average height, *h*_0_ ≈ 10 μm within the first 100 s. The approximate square root scaling of ⟨*h*⟩(*t*) is independent of substrate temperature and field of observation. We rationalize lubricant depletion in the micropillars’ interstices by the formation of wetting ridges and cloaking of the condensing droplets. The direct correlation between condensation and depletion is supported by the square root scaling of the latter, which is similar for diffusion-controlled condensation (eq 6, Methods). Furthermore, the depletion rate is proportional to the set-point temperature of the substrate (*viz*. Figure 2e), which, again, is typical for diffusion-controlled condensation, where supersaturation (or here undercooling) determines the magnitude of the condensation rate.

For hierarchically structured surfaces, the depletion dynamics changes slightly: While lubricant height evolutions show a similar dependence between set-point temperature and depletion rate (*cf*. Figure 2e), lubricant depletion deviates from the square root behavior (Figure 2f). We speculate that the delayed coalescence accompanied by a large number of smaller droplets causes the altered depletion dynamics. It should be noted that the wetting properties of the surface affects the location of preferred droplet nucleation, the number and size of condensed droplets,^25^ and therefore frost formation and propagation.

### Mesoscopic Frost Formation and Propagation

**Figure 3:**
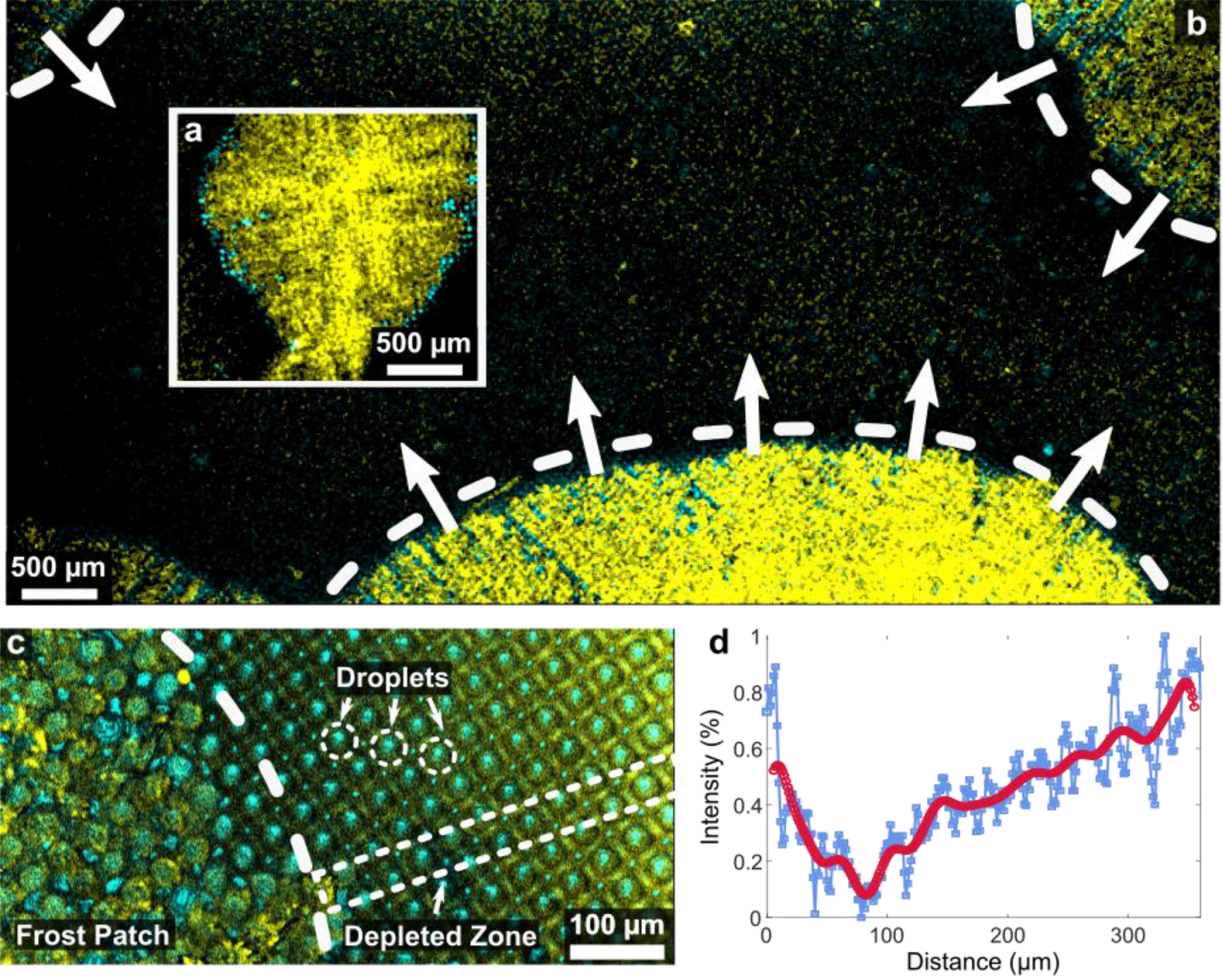
Frost formation and propagation on lubricant-infused surfaces. Frost patches form and propagate on the surface, while holding a large amount of fluorescence dyed lubricant (yellow). Simultaneously, the reflection light is monitored (cyan). (a) Macroscopic top view of a freshly formed frost patch reveals a region with increased reflection signals in the vicinity of the patch’s perimeter (cyan). Because unlubricated SU-8 (*n*_SU-8_ ≈ 1.6) reflects light better than lubricants (*n*_SiOil_ ≈ 1.4), an increased reflection signal implies a lubricant depleted zone. Outside the frost patch, lubricant appears black. (b) Frost patches propagating over the lubricant-infused micropillar array (field of view: 6440 μm × 3220 μm). The frost grows (*u*_ice_ = 1.4 ± 0.5 μm/s) from the sides into the field of view. Images were taken at a rate of 0.96 s^–1^ c). Magnified frost front (*cf*. Movie S1). Region of depletion marked in the dashed box. (d) Typical fluorescence intensity signal along the dashed box shown in Figure 3c. The blue line is the raw signal, while the red line is noise (*via* low-pass) filtered. The periodicity in the blue curve agrees with the spacing of the micropillars. The optical signals are integrated over a depth of approximately 20 μm.

At the subzero surface temperatures, prolonged exposure eventually results in frosting of condensate droplets. We observe formation and propagation of the lubricant-covered frost patches with a lower magnification objective (2.5×), providing a wider field of observation (Figure 3a,b). Placing the focal plane several μm above the micropillar base allows to accurately trace the frost. The higher focal plane also ensures that the lubricant within the micropillar array does not contribute to the integrated fluorescence intensity signal. Sometime after the initial 100 s, circular frost patches of increased fluorescence signal can be observed on the surfaces (Figure 3a). The patches propagate over the surface (Figure 3b), with an average front speed of *u*_ice_ = 1.4 ± 0.5 μm/s, Figure S6. To understand the formation and propagation of frost, we reconsider the situation where we have one large water droplet on each micropillar top (Figure S7). Freezing is initiated by the formation of stable, heterogeneous nucleation sites (ice crystals) in the liquid droplets at the pillar–liquid interface.^35^ In general, freezing is a random process due to the inherently stochastic nature of nucleation.^36^ Hence, the droplets do not freeze simultaneously, but instead in a staggered manner. Vapor pressure over liquid water exceeds that over frozen droplets by a ratio of approximately 1.1 at −12 °C.^37^ This generates a vapor flux along the vapor pressure gradient. Water vapor molecules attach at the interface of the frozen droplet toward air. Small spikes and edges on the interface (perturbations) cause the compression of the vapor field locally above the solid–vapor interface, yielding a higher supersaturation. This amplifies the growth of the small spikes and edges (Mullins–Sekerka type of instability), giving rise to the formation of frost dendrites.^38^ Ice bridges grow out from the frozen droplet and past the spacing distance of micropillars, reaching adjacent liquid droplets. Upon contact with the ice bridges, adjacent droplets freeze immediately.^22^ This process repeats, enabling a frost cluster to grow gradually.^4^

### Depletion Zones

Frost propagation is accompanied by capillary-induced suction of lubricant into the dendritic frost domains, as reflected by the high fluorescence intensity in the frost (Figure 3a,b). Further, directly in front of the propagating frost (2–3 pillars), the reflection signal (cyan) is noticeably more pronounced. This results from the pillar’s top faces that were initially coated by an approximately 1 μm-thick layer of lubricant (Figure 2a). Plain SU-8 has a higher refractive index (*n*_SU-8_ ≈ 1.6)^39^ compared to silicone oil (*n*_SiOil_ ≈ 1.4).^32^ Thus, a stronger reflection signal indicates lubricant depleted zones. Depletion zones, however, do range further than only 2–3 pillars in front of the propagating frost, as we visualize with magnified (10× objective lens) top-view recordings of the frost front (Figure 3c, Movie S1). Here, the frost propagates from the left to the right side within the field of view. To the left of the dashed white line, the irregular yellow domains reveal the frost-covered areas. To the right, the pillars are still covered by a single droplet per pillar (cyan). Figure 3d shows the average fluorescence intensity within the dashed box of panel Figure 3c. Right in front of the frost front, the integrated fluorescence intensity signal passes a minimum and then gradually rises again further ahead of the frost. This unravels the presence of depletion zones, ranging 100–200 μm, in the direction normal to the frost front. Frost propagation ends when the condensed water droplets are entirely transformed into frost. The surface is then mostly covered with frost, leaving only small uncovered islands. The final area covered by frost depends on the amount of vapor which is initially induced into the frosting chamber.

### Frost-Induced Lubricant Depletion on Microstructured LIS

**Figure 4:**
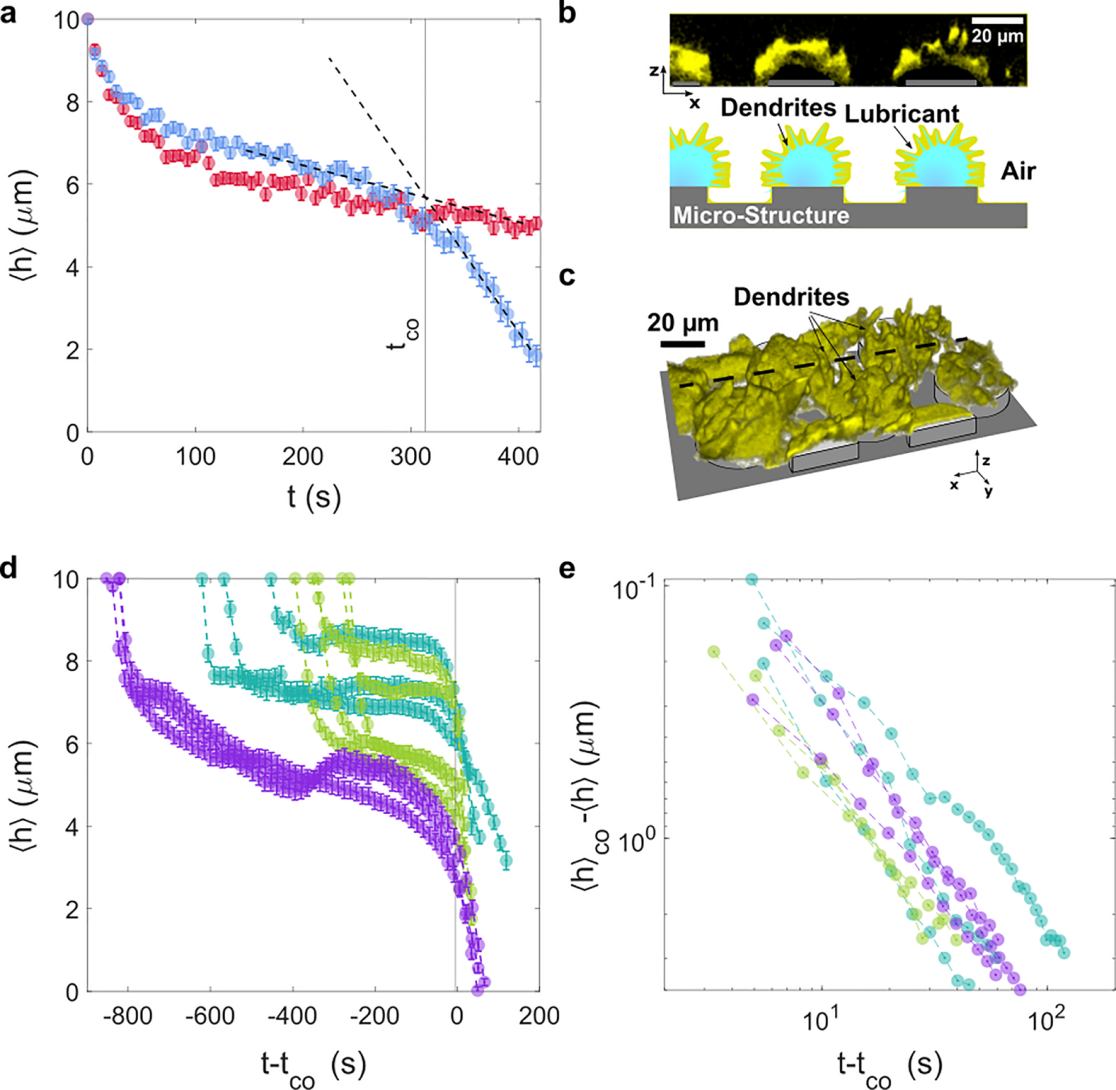
Lubricant depletion during condensation frosting. (a) Averaged height of lubricant over time during condensation frosting at −12 °C; *t* = 0 s corresponds to the beginning of observation on bare microstructured surfaces. Red and blue curves correspond to different locations of measurement on the substrate. Frost may (blue) or may not (red) propagate through the field of observation. Time, *t*_co_, refers to the onset of frost depletion by finding the linear intersection between the condensation and frosting regime (dashed lines). (b) LSCM images of vertical cross section and (c) 3D LSCM image showing frost covered by lubricant. (d) Lubricant height evolution on micro- (−22 °C, purple) and hierarchically structured surfaces (−17 °C, turquoise and −22 °C, light green). Initial offsets are due to the condensation regime. Curves are time-shifted such that *t*_co_ (crossover between condensation and frosting) coincides for all measurements; *t*_co_ depends on the location of observation, the supercooling of the surface and the humidity. (e) Lubricant reorganization during frosting as logarithmic plot, focusing on the time domain after the crossover time, *t*_co_ reveals identical dynamics.

To monitor the dynamics of lubricant depletion during frost propagation, we return to the microscopic field of observation, centered on four half-pillars (100× objective lens, 40 μm × 40 μm). On microstructured surfaces, the average lubricant height was then measured for 420 s at two characteristic locations (visualized by the red and the blue curves) (Figure 4a). One location was eventually covered with frost (blue curve), while the other remained uncovered (red curve). As the profiles of the blue and the red curve nearly overlap in the condensation phase, for *t* < 100 s, we deduce that early depletion is unaffected by the location of the field of observation. However, while the decrease of lubricant height of the red curve changes only marginally after 100 s, ⟨*h*⟩(*t*) may cross over into a steep decrease (blue curve). Considering the global process of frost propagation in Figure 3a, it becomes clear why frosting-induced depletion dynamics bifurcates into two distinctively different types, per red and blue curves. For the blue curve, the frost front approaches the field of observation, sucking the lubricant from the array into the frost dendrites. This becomes notable at the crossover time *t*_co_ ≈310 s, at which the two linear slopes (Figure 4a dashed lines) before and after crossover intersect. At the last time point shown (*t*_end_ = 420 s), the frost front arrives at the field of observation, and dendritic frost is clearly visible (*cf*. Figure 4b,c). Lubricant cloaks the frozen water, revealing the morphology of the frost features. For the red curve, only far-range lubricant depletion from a distant frost patch influenced the lubricant height. This led to significantly slower depletion dynamics. Eventually, the surface is only partially covered with frost patches because all condensed water has been converted into frost. The height depletion of the red curve stabilizes, as the field of observation resides at an uncovered region. Thus, after the condensation period of *t* > 100 s, the evolution of the average height ⟨*h*⟩(*t*) in Figure 4a depends on the field of observation, and the lubricant dynamics is governed by the proximity to frost patches.

### Frost-Induced Lubricant Depletion on Hierarchical LIS

To understand whether the two-step depletion depends on degrees of surface roughness, we compared measurements on both micro- and hierarchically structured LIS. Figure 4d,e shows the evolution of lubricant height on micro- (−22 °C, purple) and hierarchically structured surfaces (−17 °C, turquoise, and −22 °C, light green). Qualitatively, the lubricant evolution on the two surface types did not differ. However, the crossover time *t*_co_ is not identical across various measurements due to stochastic nucleation delay. After shifting the curve such that *t*_co_ coincides for all measurement, the long-term depletion dynamics follows the same power law, *h* ∼ *t*^1.27^, Figure 4e. This hints that the long-term depletion dynamics does not depend on the roughness of the micropillars. As each set point temperature experiment was conducted on the same surface, we note that the surfaces’ original filling could be restored, after removing the ice *via* melting and evaporation.

### Modeling Frosting Induced Lubricant Depletion on LIS

Next, we aim to understand the coupling between lubricant flow and frost formation/propagation. The frost dendrites (Figure 4c) form a dynamically arranging porous network into which the lubricant can wick. The wicking of the lubricant is facilitated by a higher capillary suction pressure exerted on the lubricant by the dendritic structure than that exerted by micropillars. The pressure difference due to capillary suction can be estimated as
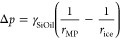
1The pressure difference is governed by the differing capillary radii of the lubricant in the micropillar array, *r*_MP_, and in the dendritic network, *r*_ice_ (eq 1). The capillary radius in the micropillar array is given by the geometry of micropillar spacing (*r*_MP_ ≈10 μm, *cf*. Methods). For an effective suction flux of lubricant into the dendritic structures, *r*_ice_ has to be smaller than *r*_MP_. The surface tension of the lubricant (silicone oil) is denoted with γ_SiOil_.

**Figure 5:**
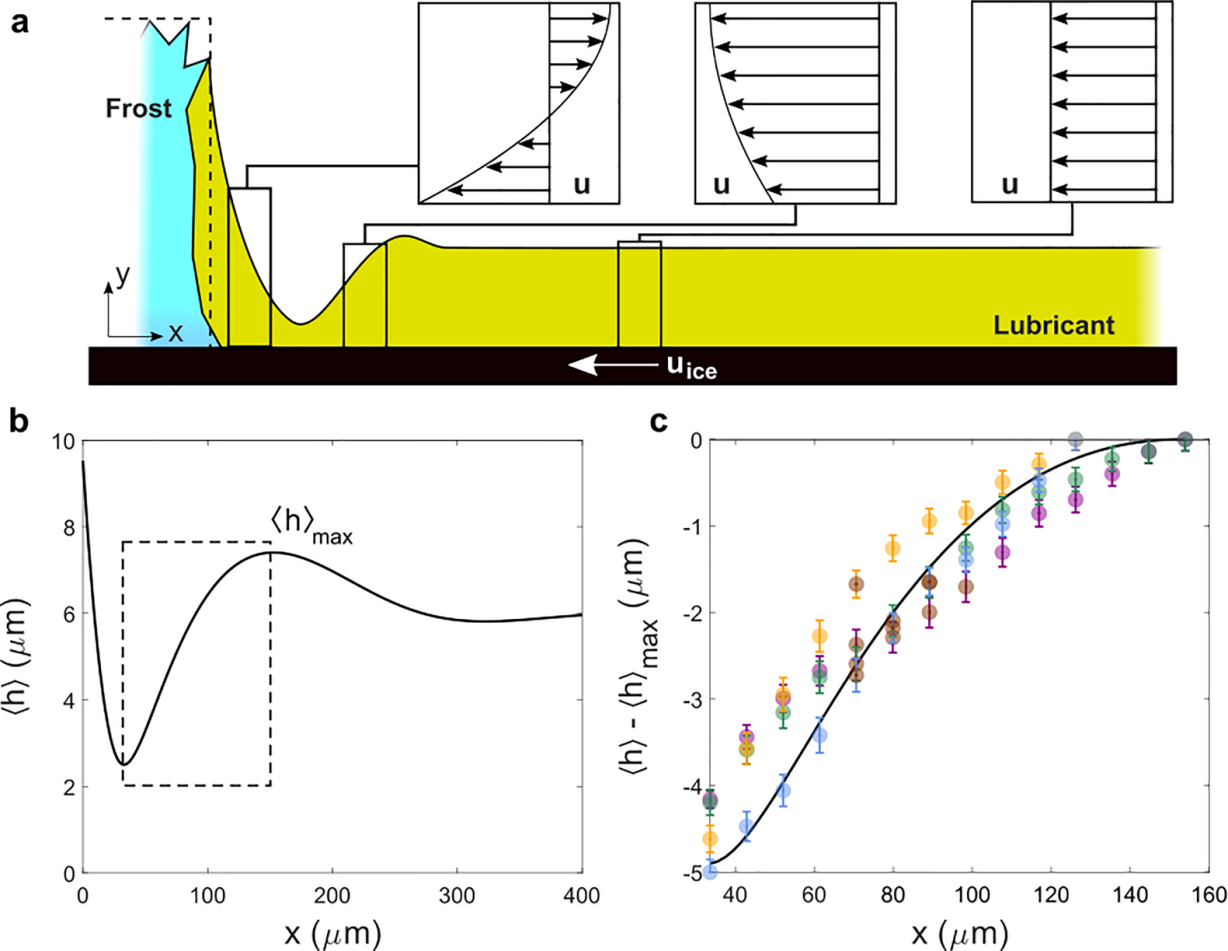
Lubricant film profile in front of the propagating frost. (a) Schematics of propagating frost front. The lubricant experiences a flow due to the suction induced by the dendritic frost domain. The insets sketch the velocity profiles at different positions. In the comoving frame, the velocity is *u* = −*u*_ice_, at the base at *y* = 0 due to no slip. (b) The lubricant height profile is calculated using the long-wave approximation. In the vicinity of the frost front, the lubricating film thins sharply, generating a depleted zone. (c) Calculated lubricant profile (solid line) in the dashed box of (b). The colored squares show repeated experimental measurements of the fast depletion process, corresponding to *t* > *t*_co_ in Figure 4. Absolute values are shown relative to ⟨*h*⟩_max_.

The suction of lubricant into the dendritic structure generates its flow in the space between the micropillars. To model lubricant dynamics, we develop a theoretical framework of the flow during the frost propagation (*cf*. SI S5). To this end, we introduce a set of coordinates with the origin at the traveling front of the frost and *x* = [0,∞) being parallel to the horizontal plane. The micropillars are not directly considered but incorporated through an increased viscosity of the lubricant (η_SiOil_ = 2.9 Pa s, *cf*. SI S4), which is, therefore, considered continuous (Figure 5a). The initial lubricant height is taken to be the same as the height of the micropillars, *h*_0_ = 10 μm. The capillary number (Ca = *u*_ice_η_SiOil_/γ_SiOil_), which relates the viscous to the interfacial forces, is based on the propagation velocity of the frost front, *u*_ice_; here, η_SiOil_ is the dynamic viscosity of the lubricant. The small capillary number, Ca ≈ 2 × 10^–4^, enables a suitable long-wave approximation^23^ for the velocity profile of the lubricant, *u*, along the vertical height *y* = [0,⟨*h*⟩], namely
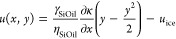
2The prefactor ∂κ/∂*x* is the average measure of the change of film curvature^40^ when crossing the frost front. Considering mass conservation yields the evolution equation for the average lubricant height perpendicular to the frost domain (*cf*. Methods):
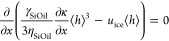
3

To determine the average height of the lubricant film, we solve eq 3 numerically (*cf*. SI S6). Computations are carried out by a well-resolved finite difference method.^41^ The number of grid points was chosen to ensure convergence. Eq 1 enters as a boundary condition at the frosting front. We set *r*_ice_ to 6 μm, which yields a good agreement between experimental observations and the numerical results. The chosen value for *r*_ice_ also aligns well with optical observations of the frost structure (Figure 4b,c). Figure 5b shows a vertical cross section of the film profile. Consistently with experimental observations (*cf*. Figure 4d), we observe a lubricant depleted zone just ahead of the frost front.

The resulting average lubricant height, together with the corresponding vertical velocity profiles at representative locations are sketched in Figure 5a and its insets. In the model, the lubricant moves toward the frost in the comoving frame with *u* = *u*_ice_ (Figure 5a), with a uniform velocity far away from the frost front. The frost domain generates a suction pressure per eq 1 due to the fine dendritic geometry. The suction into the frost domain generates a lubricant depleted zone that precedes the frost front. This results in a dimple in the overall height profile (Figure 5a,b); this dimple leads to free surface curvature gradients that affect the flow. Toward the frost front, the curvature of the dimple induces a suction pressure, resulting in a higher lubricant flow. Passing the depletion zone, the curvature becomes positive, facilitating a backflow. Therefore, the width and the height of the depletion zone are governed by two independent effects: The growth speed of the frost, *u*_ice_, and the frost suction pressure.

We discuss their respective effects on the height profile, starting with *u*_ice_. For larger values of *u*_ice_, the depletion zone is supplied by more lubricant and becomes more narrow and thicker. Additionally, the lubricant flux into the frost domain is also enhanced due to the higher availability of lubricant in the direct vicinity of the frost front. A strong suction pressure results in a deep and narrow depletion zone. Note that the suction pressure counteracts the capillary pressure within the micropillar array domain, per eq 1, so that the net lubricant flux can be tuned to effectively zero if the length scales characterizing the capillary structure of the micropillar array and the frost approach each other, that is, *r*_MP_ ≈ *r*_ice_.

To test whether the simulations quantitatively reproduce the experimental data, we compare the lubricant profiles between simulations and experiments (Figure 5c). First, we transition the experimental results to the comoving frame and define *x* = *x*_min_ + *u*_ice_ (*t*_end_ – *t*), so that *x* measures the distance from the moving front. Here, *x*_min_ is chosen as the location in the comoving frame, where the depleted zone of the numerically calculated height (*cf*. Figure 5b) is minimal. The time *t*_end_ is defined as the time at which the experimentally monitored lubricant film height is minimal (*e.g*., *t*_end_ = 420 s in the blue curve, Figure 4a). Finally, we express the results relative to the maximum height ⟨*h*⟩_max_ of the numerical solution shown in the dashed box in Figure 5b. Figure 5c plots the numerical results together with the experimental data obtained from the red curve in Figure 4a and similar curves in repeat experiments. We find excellent agreement between experimental and theoretical results, indicating that the proposed model contains all the important ingredients needed to capture the relevant physical effects.

## Conclusions

Confocal microscopy is a powerful tool for obtaining quantitative information about condensation and frost formation on lubricated surfaces, since it enables clear discrimination between the water/frost, lubricant, and the surrounding air. The formation of frost dendrites during condensation frosting induces a strong suction pressure. This leads to direct lubricant drainage and depletion. Drainage was essentially the same for micro- and hierarchically structured surfaces. Although detrimental at the first sight, we note that frost-induced lubricant depletion is reversible. Evaporation or sublimation of water restores the lubricant impregnated surface, accompanied by its characteristic features such as low friction. This is facilitated by the special properties of silicone oil, whose excellent spreading behavior often results in complete surface coverage.^17,32^ During frost formation, lubricant drainage is coupled to the speed of the dynamically forming frost front, which continuously soaks up the lubricant. These two driving mechanisms (frost propagation and capillary suction) induce lubricant depletion during frosting on liquid-infused surfaces. Interestingly, for optimally robust antifrosting surfaces, this implies a contradicting strategy: (a) Reduce frost growth speed by increasing the spacing between micropillars;^4,22,42^ and (b) increase capillary forces within the micropillar array by reducing the spacing between micropillars. We expect that such improved understanding behind the mechanism of condensation frosting on lubricated surfaces will foster the optimal design of next-generation frost-resistant lubricant-infused surfaces.

## Methods

### Fabrication of Lubricant-Infused Surfaces

The rigid micropillar surface was manufactured by spin-coating an epoxy-based SU-8 photoresist (SU-8 5, MicroChem) on a glass slide (24 × 60 mm^2^, 170 ± 5 μm thickness, Menzel-Gläser). The glass slides were cleaned by acetone and subsequently activated by oxygen plasma under 300 W for 5 min. The SU-8 photoresist was then spin-coated (500 rpm for 5 s followed by 3000 rpm for 30 s, SÜSS MicroTec) on the glass slides. The coated slides were heated at 65 °C for 3 min, 95 °C for 10 min, and then at 65 °C for 30 min, respectively. Subsequently, the samples were slowly cooled down within 2 h and exposed to UV light (mercury lamp, 350 W) under a photolithography mask for 14 s (masker aligner SÜSS MicroTec MJB3 UV400). To cross-link the photoresist, the samples were heated at 65 °C for 1 min, 95 °C for 3 min, and 65 °C for 30 min and then cooled down slowly. Next, the samples were immersed in the SU-8 developer solution for 6 min, washed with isopropanol and deionized water, and then dried in air. The micropillar array on the glass slide was cut to a circular area of approximately 3 cm^2^. Thereafter, it was plasma cleaned and infused with 0.56 μL/cm^2^ of lubricant liquid per substrate area. The wettability of flat, plasma cleaned SU-8 was measured by wetting experiments and characterized by an advancing contact angle of 12 ± 2°.

### Fabrication of Nanopatterned Hierarchical LIS

To achieve nanopatterned hierarchical LIS, an additional scale of nanoroughness was conferred to microstructured LIS. To achieve this, surface-functionalized nanoparticles were synthesized. The surface functionalization comprises two components: an epoxy terminated variant and an amine terminated variant. The epoxy terminated variant was synthesized by a methoxy-based sol–gel method, by stirring 1 g of fumed silica (Aldrich, 7 nm) in 50 mL of deionized water and 2.6 mL of (3-glycidyloxypropyl)trimethoxysilane (Aldrich, 99.9%) at 500 rpm, 20 °C, 72 h. The amine terminated variant was synthesized by an ethoxy-based sol–gel method, by stirring 1 g of fumed silica (Aldrich, 7 nm) in 50 mL of toluene (200 ppm water) and 2.8 mL of aminopropyltriethoxysilane (Aldrich, 99.9%) at 500 rpm in a round-bottom flask, under reflux at 80 °C for 72 h. Surfaces were prepared in excess reaction ratios, at 30 μmol/m^2^. Resulting colloidal solutions were then centrifuged at 10,000 rpm for 10 min and washed in their respective solvents (50 mL) for 3 cycles before being dried in a vacuum oven (50 mbar, 60 °C) overnight. Thermogravimetric analysis revealed that nanoparticles are functionalized to *ca*. 10 w/w% in both instances (amine and epoxy variants). Both nanoparticle variants were dispersed (separately) in isopropanol (2 mg/mL) by magnetic stirring (500 rpm) for 24 h, followed by ultrasonication for 1 h. The surfaces (170 μm-thick glass slides decorated with micropillars) were first cleaned *via* oxygen plasma under 120 W for 2 min. The surfaces were then dipped into the amine-functionalized nanoparticle dispersion for 10 s. The surfaces were dried in ambient air for 2 h. Thereafter, they were dipped into the epoxy-functionalized nanoparticle dispersion, again for 10 s. The hierarchically coated surfaces were then dried overnight (24 h) before use. Prior to lubricant infiltration, the surface was plasma cleaned. The wettability of flat, plasma cleaned, nanoparticle-coated SU-8 was measured by wetting experiments and characterized by an advancing contact angle of below10°.

### Lubricant and Dye

For the lubricant, we used a silicone oil (vinyl terminated polydimethylsiloxane, Gelest; surface tension: γ_SiOil_ ≈20 mN/m).^15^ A fluorescence marker (Lumogen Red F300, BASF, excitation at 532 nm, emission at 610 nm) was added to the silicone oil. To enhance the solubility of the fluorescence dye in silicone oil, the fluorescence dye was initially dissolved in chloroform (chloroform, 99.8+%, Fisher Chemical). The dye-chloroform concentration was diluted down to *c*_Lumogen Red/CHCl_3__ = 0.1 mg/mL and ultrasonicated for1 min. The dye-chloroform solution was mixed with the silicone oil such that a Lumogen Red-silicone oil concentration of *c*_Lumogen Red/SiOil_ = 0.1 mg/mL was received. The mixture was stirred for 5 min. Afterward, the mixture was exposed to 40 °C and 50 mbar for 24 h under vacuum-assisted evaporation. We did not observe changes in the interfacial tension by the dye nor an accumulation of the dye at the interfaces.

### Humidity and Temperature Control

To control the temperature of the lubricant-infused surface, the sample was mounted on a cooling element within the frosting chamber (volume: 240 mL, Figure S2, Linkam, THMS600). The cooling element is closed-loop controlled within a temperature range of −196 to 600 °C and a cooling rate up to 100 K/min. To control the chamber’s humidity, two nitrogen gas lines were used: one water vapor enriched (humidified) line and another line with dry nitrogen gas. We utilized a water-bubbler system to enrich the first gas line with water. The sample was cooled down alongside continuous purging of the chamber using the dry steam at 10 L/min. The temperature was kept at the desired set point for 5 min before commencement of the experiment. Thereafter, the flow rate of the dry nitrogen gas line was set to 4 L/min and the flow rate of the humidified gas line to 2 L/min. Both lines are connected such that the respective gas streams mix. The mixed stream was introduced into the frosting chamber for 30 s.

### Laser Scanning Confocal Microscopy

The experiments were monitored with a custom-built, inverted laser scanning confocal microscope. The microscope was controlled with a LabVIEW program. The microscope has two illuminating lasers (Cobolt DLC 25; wavelength: blue 473 nm; green 532 nm). The laser beam is sent through a magnifying objective lens to the lubricant-infused surface. The objective lens (dry; Leica HC PL FLUOTAR 2.5×/0.07; Olympus UPIanSApo 10×/0.40; Olympus LMPIanFLN 100×/0.80) of the microscope is mounted on a piezo table to translate the sample within a domain of up to 200 μm in the vertical direction. The horizontal plane is sampled with a counter rotation scanner (Cambridge Technology, 215H Optical Scanner) which sweeps with a sampling rate of 7910 ± 15 Hz in one direction. The horizontal plane of view spans an area of 40 μm × 40 μm. The observed height was 20 μm. This spatial configuration allowed a recording frequency of 6.6 Hz. The combined reflection-fluorescence modes enable discrimination between the bulk liquid lubricant phase and the surface features on the lubricant-infused surface.

### Height Extraction

The captured point cloud of the substrate from the microscope was processed with the open source package ImageJ and a custom MATLAB script. This reconstructs the spatial distribution of lubricant film (Figure S8). The lubricant height *h* is measured in the space centrically in between the micropillars. The field of observation allows a collection of 128 sampling points, which corresponds to a length of 70 μm. This corresponds to the center of one micropillar to another. The points are given with . This height profile is arithmetically averaged, which yields a scalar value, namely .

### Water Vapor Diffusion to the Substrate

The condensation of the water droplets is driven by diffusion (*cf*. SI S3). Diffusion equation of water vapor is given with
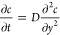
4where *c* is the water concentration, *t* is the time, and the diffusivity is given by *D*. The diffusion within the horizontal and vertical planes is considered separately, because the respective length scales differ in orders of magnitude,*O*(*l*_vert_) ≈ 10^–3^ m and *O*(l_horz_) ≈ 10^–6^–10^–9^ m. Hence, only the vertical direction *z* is considered. A solution of eq 4 is given by^43^
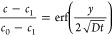
5The constants *c*_0_ and *c*_1_ are determined using the initial concentration (*c*_0_) in the whole domain, while the saturation concentration (*c*_1_) at the substrate depends on the set-point temperature. Eq 5 was fitted to the monitored relative humidity in the frosting chamber (Figure S4b). The fit revealed a diffusivity of *D* = 0.14 cm^2^/s. The condensing mass flux per area is obtained by
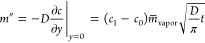
6where *m̅*_vapor_ is the the mole mass of water vapor.

### Long-Wave Approximation

We consider incompressible lubricant flow during frost propagation and fluid mechanics conservation laws (*cf*. SI S5). The flow is characterized by the capillary number (Ca ≈ 2 × 10^–4^), and the Reynolds number is based on the front propagation speed, *u*_ice_ (*Re* = ρ_SiOil_*u*_ice_*h*_0_/η_SiOil_ ≈ 2.8 × 10^–10^). For such small values of Ca and *Re*, and considering the separation of length scales in the vertical and in-plane direction, it is appropriate to consider the problem within the long-wave approach (*cf*. Kondic^44^ and references therein). This approach leads to the following equations for the depended variables (velocity (*u*,*v*)^*T*^ and pressure *p*) that in steady state read as
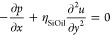
7
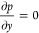
8
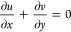
9We chose no-slip at *y* = 0 for the velocity and no-stress at *y* = ⟨*h*⟩ for the shear tensor. The pressure at *y* = ⟨*h*⟩ is given by the Laplace pressure *p* = γ_SiOil_∂κ/∂*x*. Since *p* ≠ *f*(*y*), eq 7 can be integrated twice leading to eq 2, and eq 9 gives *v*. The evolution of the average film height ⟨*h*⟩ is then given by
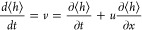
10Note that the velocities(*u*,*v*)^*T*^ in eq 9 are evaluated at *y* = ⟨*h*⟩. By introducing (*u*,*v*)^*T*^ into eq 9 and considering steady-state solutions only (∂⟨*h*⟩/∂*t* = 0), we obtain eq 3.

## Supporting Information Available

AFM roughness measurements on micropillar tops. Detailed description of experimental setup. Humidity and temperature recordings in chamber atmosphere. Raw data of 3D LSCM scans/3D image processing/laser beam influence. Measurements of area and perimeter evolution of frost patch. LSCM top-down recordings of droplet condensation on micropillar tops. Discussion on energy sinks on micropillar tops. Discussion on diffusion-driven condensation. Effective viscosity of silicone oil in micropillar array. Derivation of long-wave approximation of reorganizing silicone oil under frost formation and discretization of governing equations (PDF)
Movie S1: Top-down view of propagating frost front on lubricated impregnated microstructured surface (AVI)

## Supplementary Material

nn0c09152_si_001.pdf

nn0c09152_si_002.avi
