# Supplementary material for: How Frost Forms and Grows on Lubricated Micro- and Nanostructured Surfaces
Source: ACS Nano. 2021 Mar 1;15(3):4658–68. doi: 10.1021/acsnano.0c09152 (PMC7992192; doi:10.1021/acsnano.0c09152)
Supplement: Supplementary file 1 — nn0c09152_si_001.pdf [file nn0c09152_si_001.pdf]

How Frost Forms and Grows on Lubricated Surfaces[†]

Supplementary Information

Lukas Hauer,[‡] William S. Y. Wong,[‡] Valentina Donadei,[¶] Katharina I. Hegner,[‡] Lou Kondic,[*,§] and Doris Vollmer[*,‡]

‡*Physics at Interfaces, Max Planck Institute for Polymer Research, Ackermannweg 10, 55128 Mainz, Germany*

¶*Faculty of Engineering and Natural Sciences, Tampere University, P.O. Box 589, FI-33014 Tampere, Finland*

§*Department of Mathematical Sciences and Center for Applied Mathematics and Statistics, New Jersey Institute of Technology, Newark, NJ 07102, USA*

E-mail: kondic@njit.edu; vollmerd@mpip-mainz.mpg.de

[†]Lukas Hauer and William S. Y. Wong contributed equally

Supplementary Information

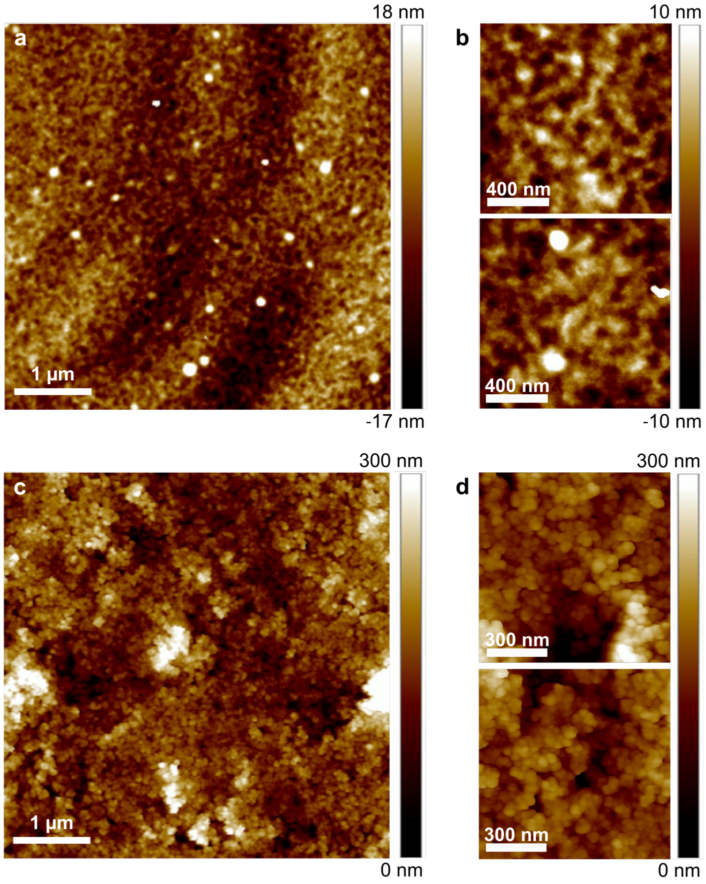

Figure S1. Roughness of bare and nanoparticle coated micropillar tops. The topography was measured with Atomic Force Microscopy (AFM, Dimension icon with ScanAsyst) in tapping mode in air. a) Bare micropillar top (field of measurement: 5 μm^2) with an average arithmetic roughness of 3.8 nm. The highest and the lowest points in this measurement are separated by 71 nm. b) AFM measurements on two additional, bare micropillar tops. The average arithmetic roughness for the top image is 2.4 nm and for the bottom 2.7 nm (field of measurement: 1 μm^2, respectively). This yields an average arithmetic roughness value of 3.4 ± 0.3 nm. c) Nanoparticle coated micropillar top (field of measurement: 5 μm^2) with an average arithmetic roughness of 36.9 nm. The highest and the lowest points in this measurement are separated by 613 nm. d) AFM measurements on two additional, nanoparticle coated micropillar tops. The average arithmetic roughness for the top image is 33.7 nm and for the bottom 27.7 nm (field of measurement: 1 μm^2, respectively). This yields an average arithmetic roughness value of 35.1 ± 1.7 nm.

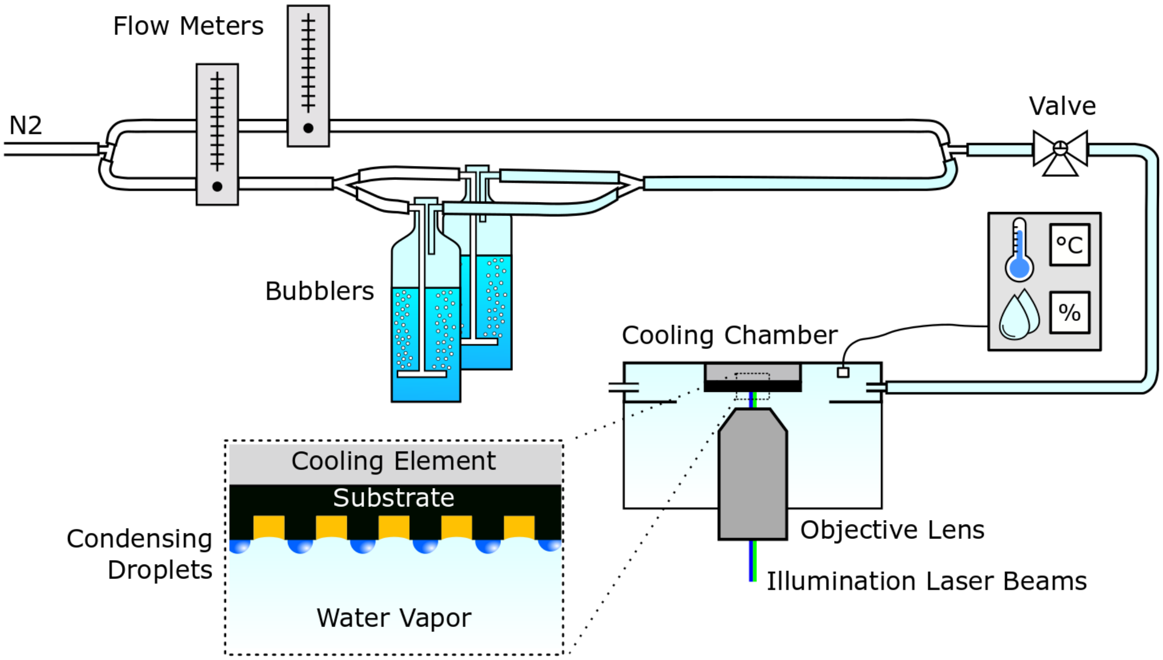

Figure S2. Experimental setup for condensation frosting. Schematics of the experimental setup. The substrate is inversely mounted on the cooling element (Linkam, THMS-600) over the objective lens of the confocal microscope. We use a blue (wavelength: 473 nm) and a green (wavelength: 532 nm) laser beam for illumination. The cooling element is controlled thermoelectrically and additionally cooled with liquid nitrogen. Dry nitrogen gas is mixed with the humidified gas stream to set the humidity in the cooling chamber. The humid atmosphere in the chamber initiates condensation of liquid water droplets on the cooled substrate.

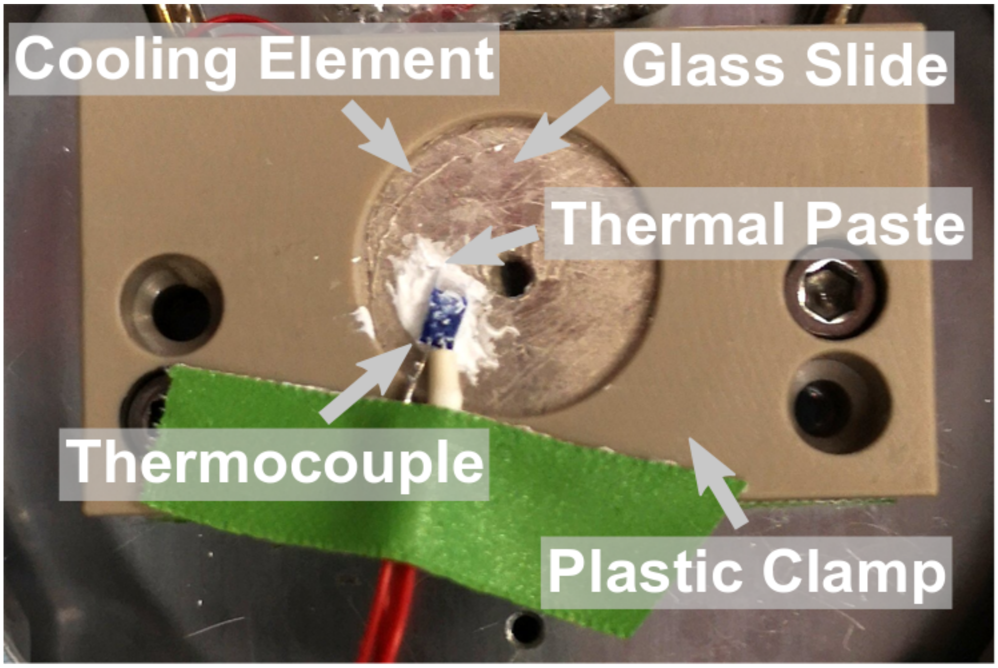

Figure S3. Calibration of the substrate temperature. To calibrate the temperature on the substrate ($T_{\text{set-point}}$), we used a glass slide. A circular glass slide was clamped to the cooling element (Linkam, THMS-600). The cooling element was cooled down from ambient temperature to -15 °C with 2 K/min. Thereafter we let the system equilibrate for 10 minutes. The temperature of the cooling element is measured internally with a thermocouple which is not visible (inside the cooling element). We stuck a second thermocouple to the top side of the glass slide with thermal paste to increase the heat conduction between the thermocouple and the glass slide. The measurement showed an offset of $T_{\text{set-point}} = -12$ °C and $T_{\text{cooling element}} = -15$ °C. This yields an offset temperature of $\Delta T = 3$ K.

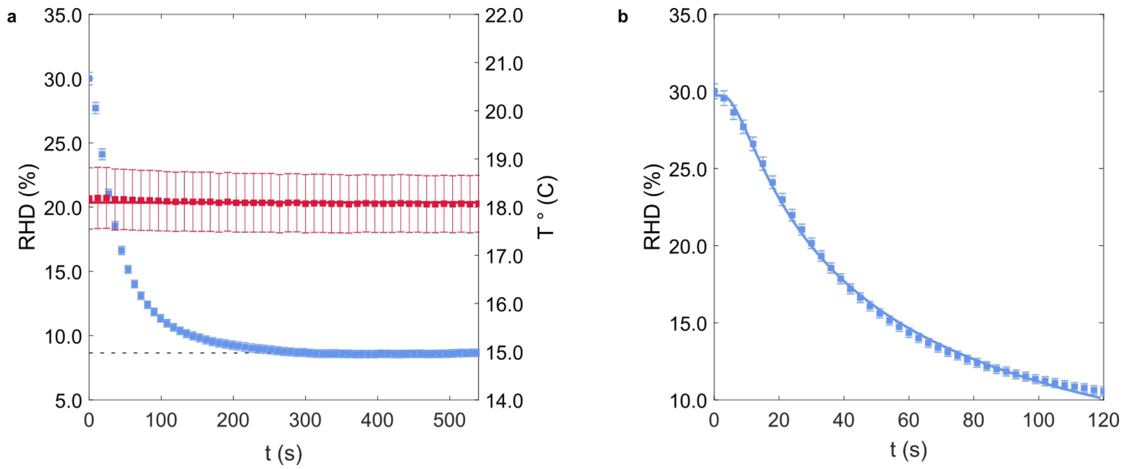

Figure S4. Relative humidity (% RHD; blue) and temperature (T; red) measurement during the experiment in the humidity chamber. The values were averaged from 14 experiment repetitions on different days.Error bars represent the relative error. The distance between the sensor and the substrate is approximately 2 cm a) Relative humidity and temperature sampling of a whole experiment. The relative humidity (blue) decays to approximately 4.0 ± 0.3 % RHD and stays constant at this value. The temperature (red) stays constant at 18.1 ± 0.6 °C (solid red line). b) Relative humidity within the first 120 s of the experiment. The measured values align well with a diffusion transport of the water vapor to the substrate (solid blue line).

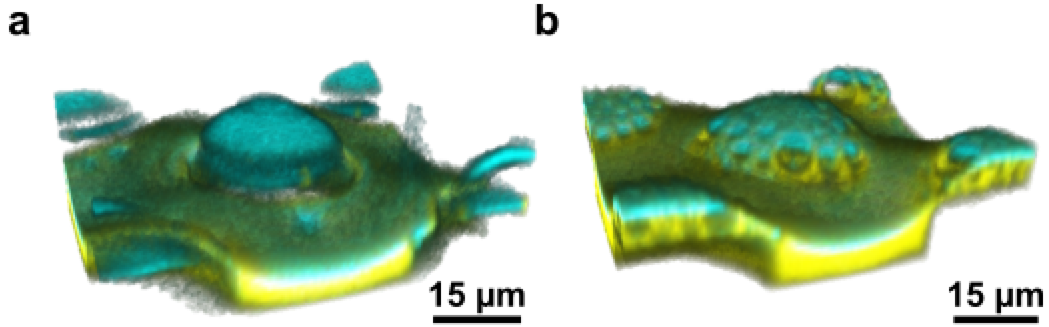

Figure S5. Raw data of Fig 2c and Fig 2d. The reflection signal appears in cyan while the fluorescence signal appears in yellow. a) Plain micropillar after humidified nitrogen carrier gas is introduced. b) Nanoparticle coated micropillar after humidified nitrogen carrier gas is introduced.

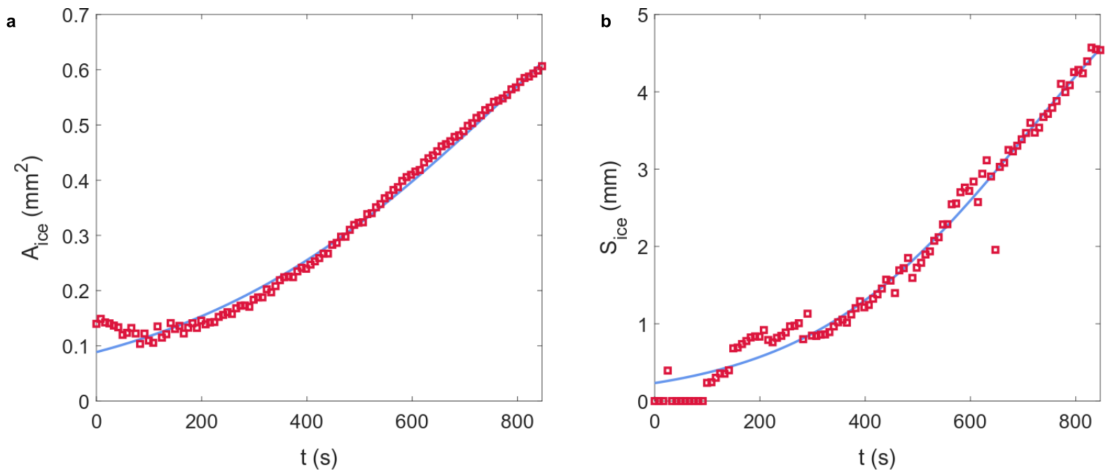

Figure S6. Area of frost patches. We monitor the frost patch evolution and extract the area and the perimeter *via* image processing. The red squares in the plot represent the measurement and the solid blue line a fitted logistic growth function. The evolution of the frost patch area A_{ice} is given by the time derivative $\dot{A}_{\text{ice}} = \oint_{S_{\text{ice}}} u \mathrm{d}S$ where u is the front propagation velocity and $\mathrm{d}S$ the line element of the perimeter S_{ice}. We define the growth speed of the frost front as the averaged velocity of the patch's propagation speed, namely $u_{\text{ice}} = \oint_{S_{ice}} u \mathrm{d}S / \oint_{S_{ice}} \mathrm{d}S$. Hence, the averaged frost front speed is $u_{\text{ice}} = \dot{A}_{\text{ice}}/S_{\text{ice}}$. By averaging u_{ice} over time and three experimental repetitions, we find $u_{\text{ice}} = 1.4 \pm 0.5$ μm/s.

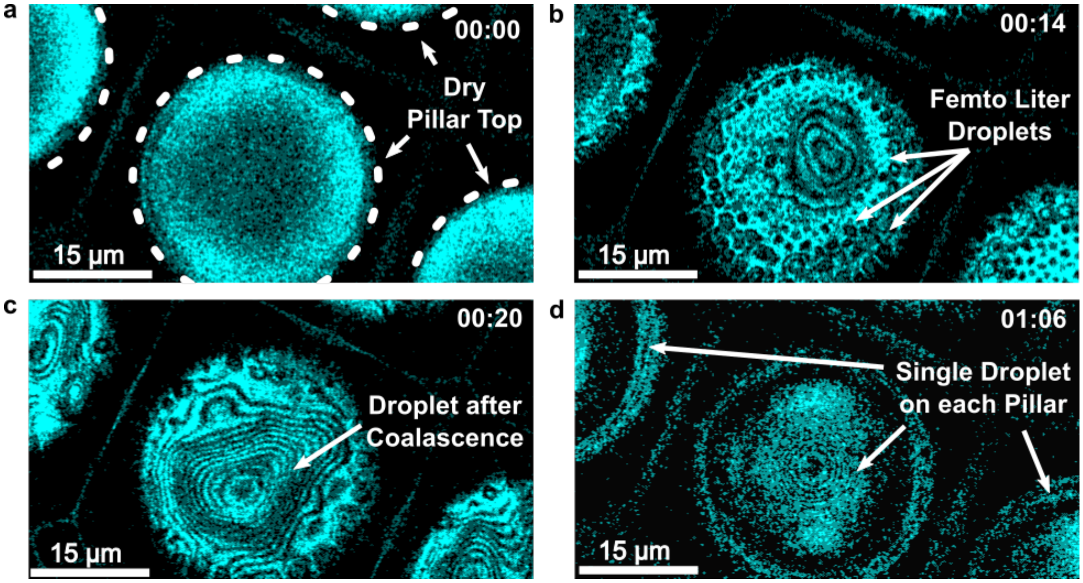

Figure S7. Reflection images of condensation of droplets on micro-pillars at −12 °C surface temperature (see also Video S1). In the upper right corner of each image, the time is given in mm:ss. We start counting the time when humidified nitrogen is introduced into the frosting chamber. a) Dry pillars. b) Shortly after the humidified nitrogen stream is introduced into the frosting chamber, femto liter droplets (diameter $\approx 1\ \mu$m) become visible on the micro-pillar tops. We are measuring in reflection mode because we cannot add a dye to condensed water. Condensed droplets appear as dark spots. c) Droplets coalesce and larger drops form. The interference pattern indicates that the droplets grew in size. d) Condensation ends with a single droplet on each micro-pillar top.

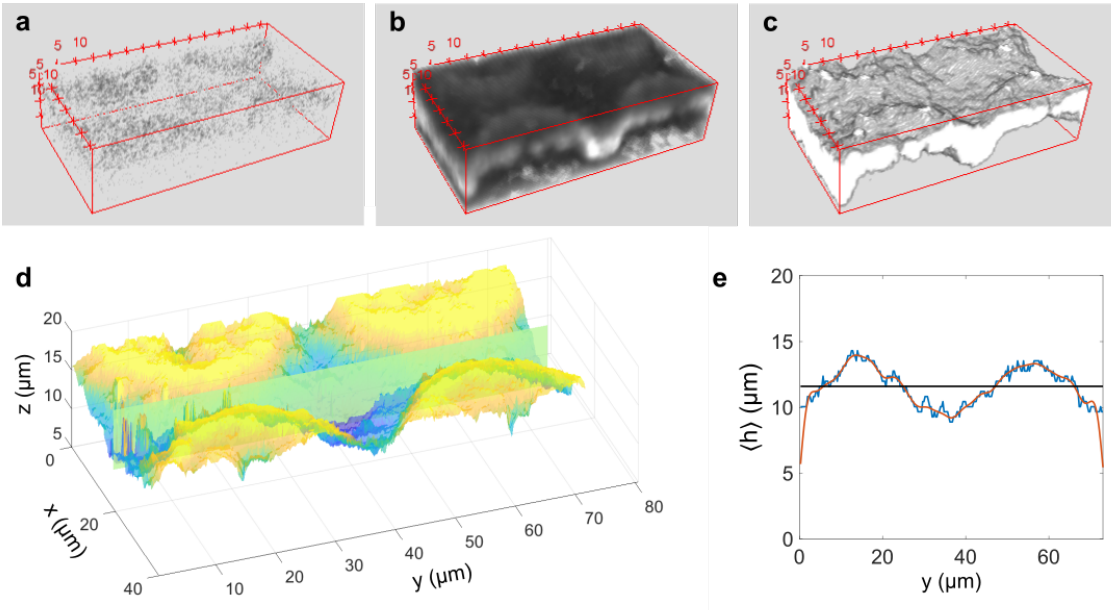

Figure S8. Image processing and height extraction of the lubricant profile. a) - c) are typical images which were preprocessed with ImageJ. a) Raw point cloud ($128 \times 128 \times 51$ pixels) of fluorescence signal. b) 3-dimensional Gauss filter with pixel radius of 6. c) Thresholding filter. d) Surface reconstruction conducted using a custom MATLAB script. The lubrication height is extracted from within the green vertical xz-plane. e) Height extraction: The blue line represents the extracted data while the orange line is the low-pass filter .

S1 Discussion on laser beam illumination

To investigate whether the laser beam induced heating affects the results we designed the following two experiments: In a first set of experiments, we conduct a typical condensation frosting experiment, Fig S9a. The surface was cooled down to -25 °C while the atmosphere was maintained dry (<5% RH, 18 °C). We introduce humidified nitrogen carrier gas for 60 seconds into the frosting cell, such that the relative humidity rises to 32%. The frost formation was recorded in a field of view of 6440 μm $\times$ 3220 μm with a 2.5x magnification lens (*cf.* manuscript). The field of view was sampled with the laser beam at a rate of 0.96s^{-1}, which corresponds to continuous illumination and is representative of the experiments. We repeated this experiment a second time were the field of observation was sampled only once every 2 minutes, leaving the surface unilluminated for the majority of time. It appears that within our experimental accuracy the formation and growth of the frost patch was identical under both conditions. The minor differences in the two experiments reflect the stochastic nature of frost formation, Fig 4 d. In a second set of experiments, we investigated the effect of laser beam illumination on melting/evaporation of the frost, Fig S9b. Therefore, we introduced a constant gas stream of dry nitrogen (room temperature) into the chamber after the frost on the surface becomes stationary. Image acquisition was conducted continuously (at a rate of 0.96s^{-1}) or only every 2 minutes while the laser was turned off between successive images. For both sampling rates the frost entirely disappeared after approximately 14 minutes. We deduce from these experiments that laser illumination does not influence the formation and growth of frost, beyond the inherent stochastics of frost. Further, the time scales for melting/evaporation of frost are unaffected.

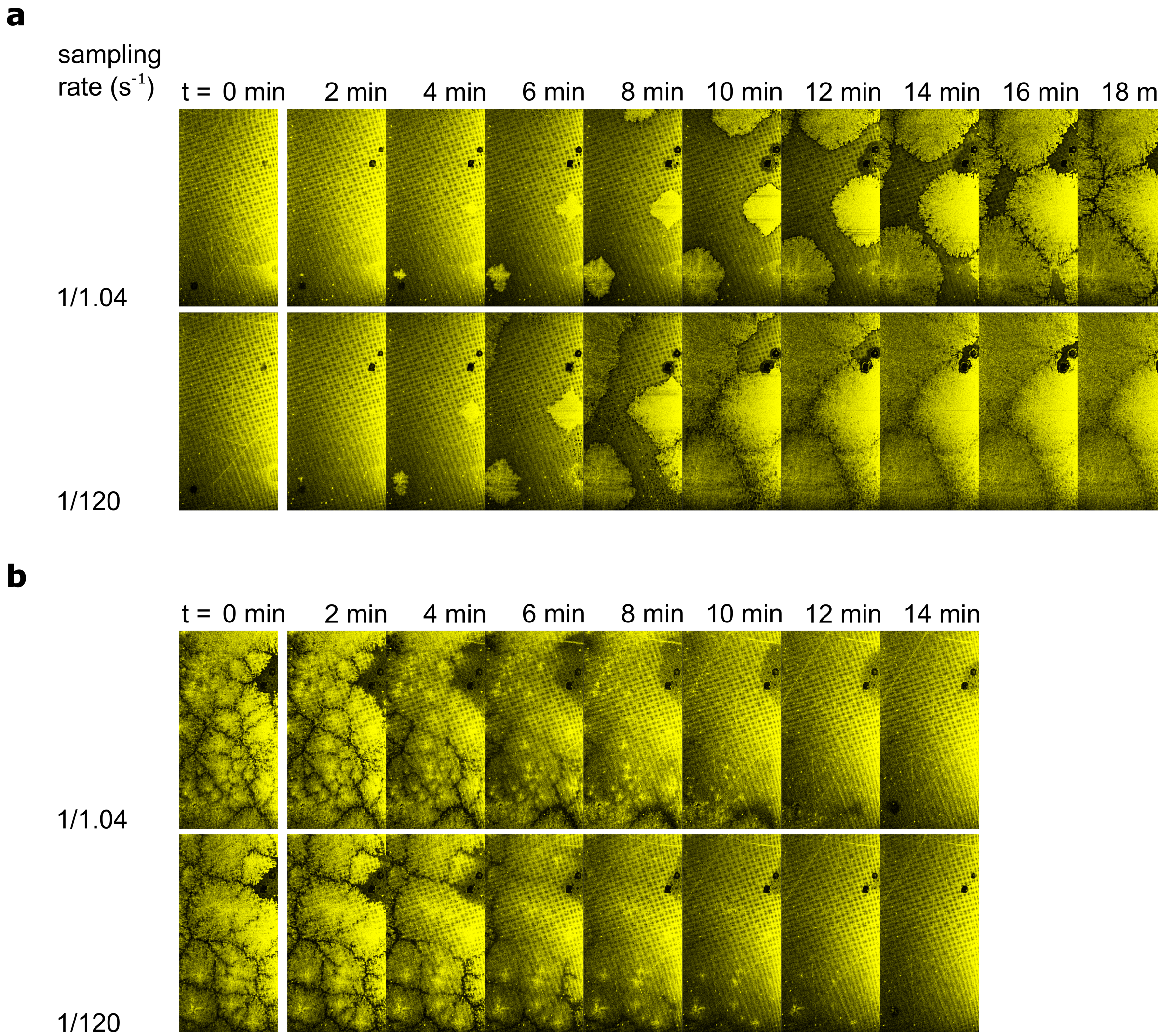

Figure S9. Influence of laser beam illumination on frosting and melting. Fluorescence microscopy of the process. The lubricant appears in yellow. Each frame is 6440 µm long and 3220 µm wide. a) Frost formation with continuous illumination (top row) and minor illumination (once every 2 minutes, bottom row). b) Melting and evaporation of frost with continuous illumination (top row) and minor illumination (once every 2 minutes, bottom row).

S2 Discussion on energy sink for nucleation

This is provided by two contributing factors. Firstly, the thermal conductivity of the rigid epoxy based SU-8 material ($\lambda_{SU-8} \approx 0.2$ W/m K)[1] is higher than the thermal conductivity of the lubricant silicone oil ($\lambda_{SiOil} \approx 0.1 - 0.2$ W/m K).[2] Hence, the surface of the micro-pillars is colder than the surface of the liquid, in between. Consequently, a higher supersaturation prevails at these locations, making nucleation more likely. Secondly, the surface energies favour condensing droplets on the pillars instead of on the lubricant for two reasons: a) The micro-pillar tops were hydrophilized prior to infiltration, lowering the surface energy, γ_{sl}, between the water nucleus and the solid SU-8 surface; and b) the thin lubricant layer on the micro-pillar tops facilitates a lower interfacial energy, γ_{lv}, between the nucleus and the surrounding lubricant (silicone oil). Hence, the required critical free energy for stable nucleus formation at the pillar's top face is significantly reduced

S3 Discussion of governing mechanisms responsible for vapor transport to the substrate

Water vapor diffuses from the supersaturated atmosphere to the substrate. Additionally, the vertical temperature gradient between the substrate and the atmosphere may induce natural advection currents of the water vapor.[3] To investigate whether diffusion or advection processes govern the vapor transport, we calculate the Rayleigh number, Ra, which represents a special form of the Péclet number. The Péclet number compares advection fluxes to diffusive fluxes, while the Rayleigh number does the same but assumes the special case of advection flux, namely gravity driven buoyancy. The Rayleigh number is defined as

$$\mathrm{Ra} = \frac{\Delta\rho g l^3}{\eta D}. \tag{1}$$

The density difference between the far-field atmosphere ($T_\infty = 18$ °C) and the substrate ($T_{\mathrm{set-point}} = -12$ °C) was calculated using the ideal gas law and a relative humidity of 30 %. This yields a density difference of $\Delta\rho = 0.139$ kg/m^3. The acceleration due to gravity is $g = 9.81$ m/s^2. $\eta = 1.75 \times 10^{-5}$ Pa s and $D = 0.247$ cm^2/s[4] are the dynamic viscosity of humid air and the diffusivity of water in nitrogen, respectively. The characteristic length scale is set as the distance between the substrate and the objective lens $l \approx 2$ mm. This leads to a Rayleigh number of Ra ≈ 25. Therefore, temperature gradient induced advection can be ignored.[5]

S4 Discussion on viscosity

The viscosity in a silicone oil bulk flow at $T = 25$ °C is given with the reference viscosity $\eta_{\mathrm{SiOil,ref}} = 194$ mPa s (200 cSt, vinyl terminated polydimethylsiloxane, Gelest). We investigate the lubricant flow in a micro-pillar array at $T = -12$ °C. Due to the temperature dependency of the viscosity,[6] we expect a value different from $\eta_{\mathrm{SiOil,ref}}$ for our considered case. Furthermore, the presence of the micro-pillars imposes additional shear friction compared to a free bulk flow (without micro-pillars). Instead of considering complex flow in micro-pillar geometry, we instead consider simpler version of a channel flow, as described in what follows. As a final result, we obtain the the effective viscosity, which includes the modifications due to a) the low temperature, with the temperature factor $\alpha\,(T = -12\ ^\circ\mathrm{C})$ and b) the imposed friction by the micro-pillars with the geometrical factor β_{geo}. Both factors together yield the effective viscosity

$$\eta_{\mathrm{SiOil}} = \alpha\,(T)\,\beta_{\mathrm{geo}}\eta_{\mathrm{SiOil,ref}}. \tag{2}$$

The temperature factor is given[7] by $\alpha\,(T = -12\ ^\circ\mathrm{C}) = 2.9$.

In order to find the geometrical factor β_{geo}, we consider the viscous pressure drops, $\Delta p^{I}_{\mathrm{vis}}$ and $\Delta p^{II}_{\mathrm{vis}}$, in in a free bulk flow, and in the flow with micro-pillars, respectively. We start

by discussing the free bulk flow. Assume a Poisson flow of silicone oil restricted by two side walls, a base and a free interface at the top, Fig S10 a,b. The side walls have a height of H and a pitch distance from one wall to the other of D. The silicone oil has a volume flux Q which flows in the y-direction. The viscous pressure drop can be calculated as[8]

$$\Delta p^{I}_{\text{vis}} = L\frac{Q}{DH}\frac{3\eta_{\text{SiOil,ref}}}{H^2}\left[1-\sum_{m=1,odd}^{\infty}\frac{1}{m^5}\frac{384}{\pi^5}\frac{H}{D}\tanh\left(m\pi\frac{D}{4H}\right)\right]^{-1}. \tag{3}$$

The length over which the pressure drops is denoted by L, and m is the m-th Fourier mode of the lubricant velocity. Note that for $H/D \ll 1$, the side walls have negligible effect on friction within the flow. In this case, the free bulk flow on a plane is recovered, and the pressure drop reads

$$\Delta p^{I}_{\text{vis}} = L\frac{Q}{DH}\frac{3\eta_{\text{SiOil,ref}}}{H^2}. \tag{4}$$

Now, we introduce obstacles into the flow domain and compare the viscous pressure drop in this new situation. In the experimental system, the obstacles are represented by the micro-pillar array. Here, we idealize the experimental system and approximate the micro-pillars by n channels, separated by walls, Fig S10 c. The height of the introduced walls is the same as of the side walls. We chose $H = 10\ \mu$m, motivated by the micro-pillar height. The pitch distance between the walls is set to $D/n = d = 10\ \mu$m, which coincides with the distance between the micro-pillars. The volume flux in a channel i is Q_i and the pressure drop is

$$\Delta p^{II}_{\text{vis}} = L\frac{Q_i}{dH}\frac{3\eta_{\text{SiOil,ref}}}{H^2}\left[1-\sum_{m=1,odd}^{\infty}\frac{1}{m^5}\frac{384}{\pi^5}\frac{H}{d}\tanh\left(m\pi\frac{d}{4H}\right)\right]^{-1}. \tag{5}$$

Finally we compare the total pressure drop, $\Delta p^{II}_{\text{vis}}$, of the system with obstacles to the pressure drop of the system without obstacles (with $H/D \ll 1$) to find the geometrical factor. We constrain the volume flux by specifying $nQ_i = Q$. The geometrical factor reads

$$\beta_{\text{geo}} = \frac{\Delta p^{II}_{\text{vis}}}{\Delta p^{I}_{\text{vis}}} = 5.8 \tag{6}$$

Note that β_{geo} is independent of the channel length, L, and only depends on the ratio d/H. Introducing $\alpha\,(T = -12\ °\mathrm{C})$ and β_{geo} into Eq. (2), we find a dynamic viscosity $\eta_{\mathrm{SiOil}} = 3.2$ Pa s. We point out that this value for the effective viscosity still overestimates the real viscosity because we approximate the micro-pillar array by a line of channels. This remark is supported by the fact that we obtained optimal match between experimental data and simulation with an effective viscosity of $\eta_{\mathrm{SiOil}} = 2.9$ Pa s.

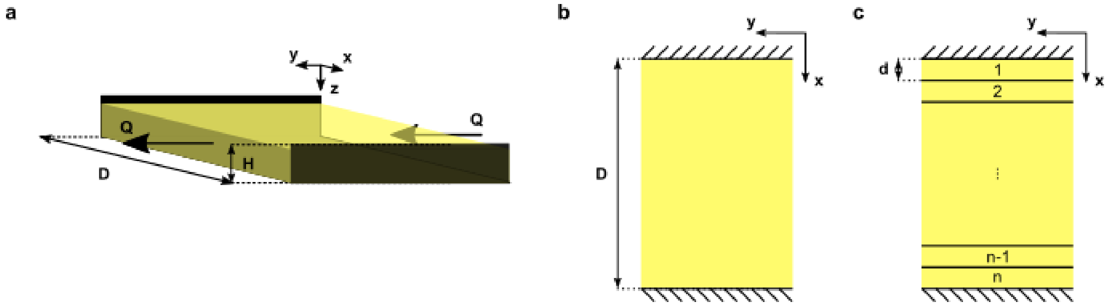

Figure S10. Schematic of silicone oil flow. a) Silicone oil flows with a volume flux Q in the y-direction. The flow is restricted by side walls of height $H = 10\ \mu$m. b) Top view of the flow domain. The two side walls are separated by a distance D. c) The experimental system that includes a micro-pillar array is idealized by channels of the same width as the pitch distance between the micro-pillars, $d = 10\ \mu$m

S5 Lubricant reorganization during condensation frosting: long-wave approximation

In the following, we report the theoretical framework that we use to model the lubricant flow. We start by discussing a general form of the conservation equation of mass and momentum,[9] followed by the discussion of the appropriate boundary conditions. By nondimensionalizing the governing equations, we identify the dominant terms, and then formulate an asymptotic description of the flow problem. We introduce a standard finite difference discretization scheme for the derived governing equation in order to find the lubricant film height. Note that the notation slightly deviates from the one utilized in the main text of the manuscript. For clarity, we start by defining the nomenclature used in this section.

S5.1 Nomenclature

Properties with physical dimension are marked with a tilde.

Position:	$[\tilde{x}, \tilde{y}]^T$
Velocity:	$[\tilde{u}, \tilde{v}]^T$
Lubricant height:	$\tilde{h}$
Density:	ρ
Dynamic viscosity:	η
Kinematic viscosity:	ν
Perturbation wave length:	λ
Initial lubricant height:	h_0
Frost propagation speed:	$\tilde{u}_{\text{ice}}$
Differential operators (Φ is a general property)	
Derivative ($\tilde{x}$ is chosen as an example):	$\frac{\partial \Phi}{\partial \tilde{x}}$ or $\partial_{\tilde{x}}\Phi$ or $\Phi_{\tilde{x}}$ Nabla operator:
$\nabla = [\frac{\partial}{\partial \tilde{x}}, \frac{\partial}{\partial \tilde{y}}]^T$ Total time derivative:	$\frac{d\Phi}{d\tilde{t}} = \frac{\partial \Phi}{\partial \tilde{t}} + \tilde{u}\frac{\partial \Phi}{\partial \tilde{x}} + \tilde{v}\frac{\partial \Phi}{\partial \tilde{y}}$

S5.2 Domain

We introduce a set of coordinates and fix the origin in the vertical direction at the base and in the horizontal direction at the frost front, as illustrated in Fig S11.

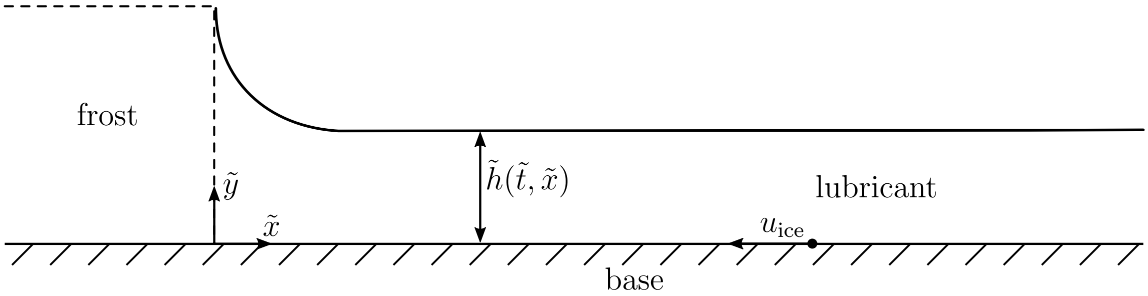

Figure S11. Schematics of the flow domain. Coordinate system is fixed at the frost front.

S5.3 Governing equations; general form

Mass conservation:

$$\frac{\partial \rho}{\partial \tilde{t}} + \frac{\partial \left(\rho \tilde{u}\right)}{\partial \tilde{x}} + \frac{\left(\partial \rho \tilde{v}\right)}{\partial \tilde{y}} = 0 \ . \tag{7}$$

For an incompressible fluid, such as the lubricant, the density ρ is constant, and (7) becomes

$$\frac{\partial \tilde{u}}{\partial \tilde{x}} + \frac{\partial \tilde{v}}{\partial \tilde{y}} = 0 \ . \tag{8}$$

Momentum conservation in the x-direction gives

$$\rho \left(\frac{\partial \tilde{u}}{\partial \tilde{t}} + \tilde{u}\frac{\partial \tilde{u}}{\partial \tilde{x}} + \tilde{v}\frac{\partial \tilde{u}}{\partial \tilde{y}} \right) = -\frac{\partial \tilde{p}}{\partial \tilde{x}} + \eta \left(\frac{\partial^2 \tilde{u}}{\partial \tilde{x}^2} + \frac{\partial^2 \tilde{u}}{\partial \tilde{y}^2} \right) \ . \tag{9}$$

Momentum conservation in the y-direction gives

$$\rho \left(\frac{\partial \tilde{v}}{\partial \tilde{t}} + \tilde{u}\frac{\partial \tilde{v}}{\partial \tilde{x}} + \tilde{v}\frac{\partial \tilde{v}}{\partial \tilde{y}} \right) = -\frac{\partial \tilde{p}}{\partial \tilde{y}} + \eta \left(\frac{\partial^2 \tilde{v}}{\partial \tilde{x}^2} + \frac{\partial^2 \tilde{v}}{\partial \tilde{y}^2} \right) \ . \tag{10}$$

The location of the lubricant height $\tilde{h}(\tilde{x}, t)$ is given by the condition

$$\tilde{h}(\tilde{t}, \tilde{x}) = \tilde{y} \ . \tag{11}$$

The evolution of lubricant height is obtained by applying the total time derivative $d/d\tilde{t}$ to Eq. (11) (at $\tilde{y} = \tilde{h}$):

$$\frac{\partial \tilde{h}}{\partial \tilde{t}} + \tilde{u}\frac{\partial \tilde{h}}{\partial \tilde{x}} = \tilde{v} \ . \tag{12}$$

S5.4 Boundary conditions

At $\tilde{x} = 0$, the stresses in the lubricant and in the frost are continuous:

$$-\tilde{p} + \eta\frac{\partial \tilde{u}}{\partial \tilde{x}} = -\tilde{p}_{\text{ice}} \ . \tag{13}$$

For $\tilde{x} \to \infty$, the stress in the lubricant vanishes:

$$-\tilde{p} + \eta\frac{\partial \tilde{u}}{\partial \tilde{x}} = 0 \ . \tag{14}$$

At $\tilde{y} = 0$, the lubricant satisfies the no-slip and no-penetration boundary condition:

$$\tilde{u} = -\tilde{u}_{\text{ice}}, \ \tilde{v} = 0 \ . \tag{15}$$

At $\tilde{y} = \tilde{h}$ the shear stress of the lubricant becomes zero and the pressure is determined by the Laplace-Young boundary condition specifying pressure jump at the interface[10]

$$\eta\frac{\partial \tilde{u}}{\partial \tilde{y}} = 0, \ \tilde{p} = -\gamma\tilde{\kappa} \tag{16}$$

S5.5 Interfacial curvature

The curvature at the interface $\tilde{y} = \tilde{h}$ is determined[11] by $\tilde{\kappa} = \nabla \cdot \mathbf{n}_\Sigma$, with $\mathbf{n}_\Sigma$ being the normal vector at the interface. The curvature becomes

$$\tilde{\kappa} = \frac{\partial_{\tilde{x}\tilde{x}}\tilde{h}}{\left(1 + \left(\partial_{\tilde{x}}\tilde{h}\right)^2\right)^{3/2}} \tag{17}$$

S5.6 Capillary pressure in the ice domain

$$\tilde{p}_{\text{ice}} = -\frac{\gamma}{R_{\text{ice}}} \tag{18}$$

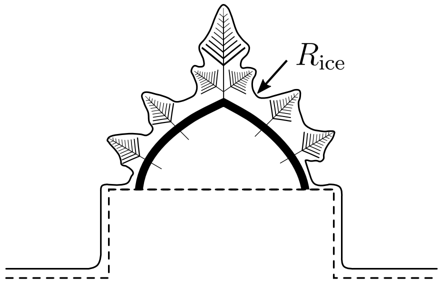

Figure S12. Lubricant film covering frozen drop and dendrites. Frozen drop sits on top of a micro-pillar.

S5.7 Dimensionless variables

We nondimensionalize the variables as follows:

$$x \equiv \tilde{x}/\lambda,\ y \equiv \tilde{y}/h_0,\ t \equiv \tilde{t}\tilde{u}_{\text{ice}}/\lambda,\ h \equiv \tilde{h}/h_0,$$

$$\epsilon \equiv h_0/\lambda,\ u \equiv \tilde{u}/\tilde{u}_{\text{ice}},\ v \equiv \epsilon\tilde{v}/\tilde{u}_{\text{ice}},\ p \equiv \tilde{p}\epsilon^{-1}\lambda/\gamma$$

$$\text{Ca} = \frac{\tilde{u}_{\text{ice}}\eta}{\gamma}, \qquad \text{Re} = \frac{\tilde{u}_{ice}h_0}{\nu}\ .$$

Here, Ca and Re are the capillary number and the Reynolds number, respectively. We define a horizontal scale λ, utilizing the capillary number, as

$$\lambda = h_0 \left(3\mathrm{Ca}\right)^{-1/3} . \tag{19}$$

Eq. (19) relates $\epsilon = h_0/\lambda$ with the capillary number Ca; note that $\mathrm{Ca} \sim \mathcal{O}\left(\epsilon^3\right)$.

S5.8 Non-dimensional governing equations

Mass conservation:

$$\frac{\partial u}{\partial x} + \frac{\partial v}{\partial y} = 0 . \tag{20}$$

Momentum conservation, x-direction:

$$\epsilon \mathrm{Re} \left(\frac{\partial u}{\partial t} + u \frac{\partial u}{\partial x} + v \frac{\partial u}{\partial y} \right) = -3 \frac{\partial p}{\partial x} + \epsilon^2 \frac{\partial^2 u}{\partial x^2} + \frac{\partial^2 u}{\partial y^2} . \tag{21}$$

Momentum conservation, y-direction:

$$\epsilon^3 \mathrm{Re} \left(\frac{\partial v}{\partial t} + u \frac{\partial v}{\partial x} + v \frac{\partial v}{\partial y} \right) = -3 \frac{\partial p}{\partial y} + \epsilon^2 \left(\epsilon^2 \frac{\partial^2 u}{\partial x^2} + \frac{\partial^2 u}{\partial y^2} \right) . \tag{22}$$

Evolution of lubricant height ($y = h$):

$$\frac{\partial h}{\partial t} + u \frac{\partial h}{\partial x} - v = 0 . \tag{23}$$

Interfacial curvature:

$$\kappa = \frac{\partial_{xx} h}{\left(1 + \epsilon^2 \left(\partial_x h\right)^2\right)^{3/2}} . \tag{24}$$

S5.9 Long-wave approximation for $\epsilon^2, \epsilon \mathrm{Re} \ll 1$

Momentum conservation, x-direction:

$$\frac{\partial^2 u}{\partial y^2} = 3\frac{\partial p}{\partial x} \ . \tag{25}$$

Momentum conservation, y-direction:

$$0 = \frac{\partial p}{\partial y} \ . \tag{26}$$

Interfacial curvature:

$$\kappa = \frac{\partial^2 h}{\partial x^2} \ . \tag{27}$$

The mass conservation, Eq. (20) and the evolution equation for the film height, Eq. (23) preserve their respective form. Figure S13 shows the resulting domain in terms of nondimensional variables.

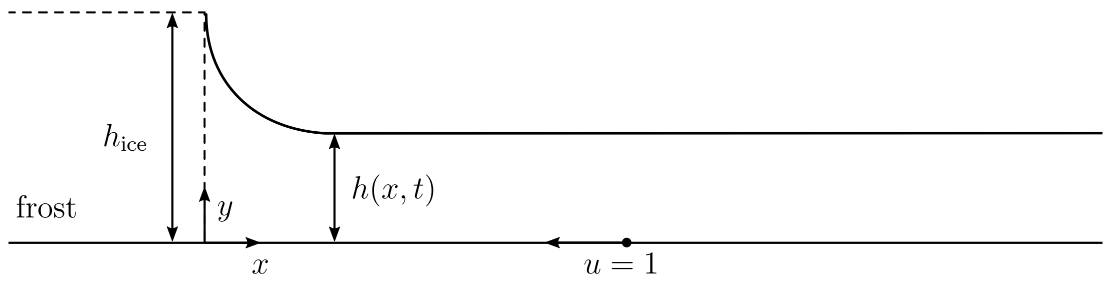

Figure S13. Considered domain in terms of nondimensional variables.

S5.10 Thin film equation

The velocities u and v at $y = h$ can be found by integrating equations (20), (25) and (26). Together with the no-slip and no-penetration condition at the base, the no-shear condition, and the Laplace-Young condition at $y = h$, we find

$$u(y = h) = \frac{3}{2}\frac{\partial \kappa}{\partial x}h^2 - 1 \ , \tag{28}$$

$$v(y=h) = -\left[\frac{\partial^2 \kappa}{\partial x^2} h^3 + \frac{1}{2}\frac{\partial \kappa}{\partial x}\frac{\partial h}{\partial x} h^2\right] \; . \tag{29}$$

Inserting equations (28)- (29) into Eq. (23), we obtain the evolution equation for the film height, h,

$$\frac{\partial h}{\partial t} + \frac{\partial}{\partial x}\left[\frac{\partial \kappa}{\partial x} h^3 - h\right] = 0 \; . \tag{30}$$

S5.11 Boundary conditions

Utilizing the boundary conditions (13) and (14), yields

at $x = 0$:

$$\frac{\partial^2 h}{\partial x^2} + \left(\frac{\mathrm{Ca}^2}{3}\right)^{1/3} \frac{\partial u}{\partial x} = \frac{h_0}{R_{\mathrm{ice}}} \left(3\mathrm{Ca}\right)^{-2/3} \; , \tag{31}$$

and for $x \to \infty$:

$$\frac{\partial^2 h}{\partial x^2} + \left(\frac{\mathrm{Ca}^2}{3}\right)^{1/3} \frac{\partial u}{\partial x} = 0 \tag{32}$$

Since $\mathrm{Ca}^2 \ll 1$, the second term on the left hand side of Eq. (31) (due to the normal compression) can be neglected. For closure, two further boundary conditions are required. Since the lubricant wets the ice, it is reasonable to assume a film height which matches the height of the ice front h_{ice} at $x = 0$. Since there is no inflow for $x \to \infty$, we specify zero flux there. To summarize, the four boundary conditions read as follows:

at $x = 0$:

$$h = h_{\mathrm{ice}} \; , \tag{33}$$

$$\frac{\partial^2 h}{\partial x^2} = \frac{h_0}{R_{\text{ice}}} (3\text{Ca})^{-2/3} \ , \tag{34}$$

and at $x \to \infty$:

$$\frac{\partial^3 h}{\partial x^3} = 0 \ , \tag{35}$$

$$\frac{\partial^2 h}{\partial x^2} = 0 \ . \tag{36}$$

S5.12 Initial condition

Eventually we are only interested in the steady state solution of Eq. (30. However, in order to solve this equation, we need an initial condition. Hence, we choose an arbitrary function for $h(t = 0)$ which satisfies the boundary conditions. We use

$$h(x) = h_{\text{ice}} \left(\alpha + (\alpha - 1)e^{-x\beta} \right) \ , \tag{37}$$

where $\alpha \in (0, 1]$ and

$$\beta = \sqrt{\frac{\lambda}{\epsilon R_{\text{ice}} \alpha h_{\text{ice}}}} \ .$$

S6 Discretization

S6.1 Space domain

The flux term in Eq. (30) is discretized using a standard finite difference scheme.[12] The grid points are equally spaced, with a spacing of $L/N = \Delta x$, and the physical domain is specified by $x \in [0, L]$. The grid points lie within the computation domain and are defined starting at $\Delta x/2$:

$$x_i = i \cdot \Delta x - 0.5\Delta x \quad i \in [1, N+1]. \tag{38}$$

The film height and the derivatives needed are specified as follows:[13]

$$h(x_i - 0.5\Delta x) \approx h_{1,i} = \frac{h(x_i) + h(x_{1+i})}{2} \ , \tag{39}$$

$$h_{xx}(x_i) \approx h_{xx,i} = \frac{h(x_{i+1}) - 2h(x_i) + h(x_{i-1})}{\Delta x^2} \ , \tag{40}$$

$$h_{xxx}(x_i - 0.5\Delta x) \approx h_{xxx,i} = \frac{h_{xx,i+1} - h_{xx,i}}{\Delta x} \ . \tag{41}$$

The approximated flux term, j_i, becomes

$$j_i = h_{xxx,i} \left(h_{1,i}\right)^3 - h_{1,i}. \tag{42}$$

Finally, the derivative of the flux is calculated as

$$dj_i = \frac{j_{i+1} - j_i}{\Delta x}. \tag{43}$$

S6.2 Discrete boundary conditions

In order to describe the fluxes we need additional points, outside of the computational domain. We do this by introducing two (ghost) points on both sides: $\{i \in W_{\setminus 0} | i = [1, N] \cup [-2; -1; N+2; N+3]\}$. On these points we need to find values of h_1, which is possible by utlizing the boundary conditions, equations (33) - (36). We obtain a set of equations, describing h_1 in the ghost field

$$h(x = 0) = h_{\text{ice}} \approx h_{1,1} \ , \tag{44}$$

and

$$h_{xx}(x=0) = \frac{h_0}{R_{\text{ice}}} (3\text{Ca})^{-2/3} \approx \frac{h_{xx,-1} + h_{xx,1}}{2} \; . \tag{45}$$

Note that $h_{xx,-1}$ and $h_{xx,1}$ are calculated using Eq. (40), and that calculation of $h_{xx,-1}$ involves the $i = -2$ ghost point. The points at the right boundary are linked to the boundary condition by

$$h_{xxx}(L) = 0 \approx h_{xxx,N+1} \tag{46}$$

and

$$h_{xx}(L) = 0 \approx \frac{h_{xx,N} + h_{xx,N+1}}{2}. \tag{47}$$

$h_{xxx,N+1}$ is calculated using Eq. (41). Note that its calculation requires the use of the $N+3$ ghost point.

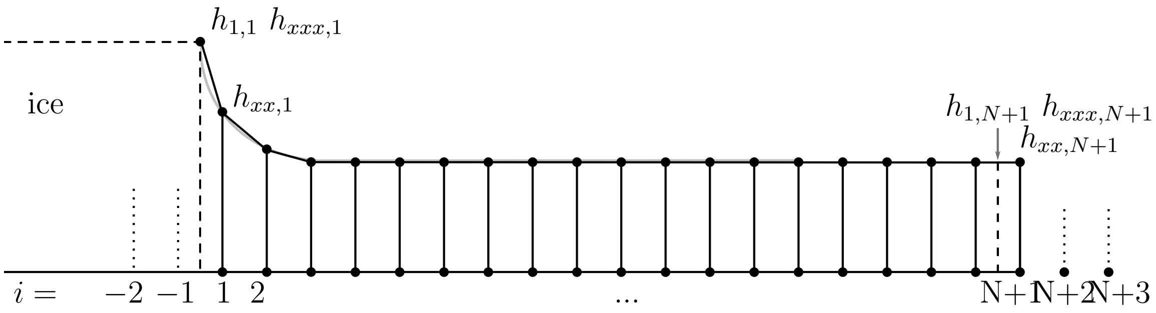

Figure S14. Spatial discretization with N points and 4 ghost points.The vertical dashed line to the left corresponds to $x = 0$ and the one to the rigth to $x = L$.

S6.3 Time domain

The continuous time, discrete space form of Eq. (30) reads

$$\frac{dh}{dt} \approx dj_i. \tag{48}$$

for $t \in [t_0, t_f]$. The time derivative is discretized using numerical differentiation formulae (NDF).[14] This method provides good convergence for stiff ODE systems.

S6.4 Steady state

To consider the steady state solution of the discretized Eq. (30), we evaluate the change between two subsequent discrete time steps $k-1$ and k,

$$\epsilon_k = dj_{k\,2} - dj_{k-1\,2}\,. \tag{49}$$

with $dj_{k\,2}$ being the L2-norm of the k-th time step. We define the steady state for $\epsilon_{k\,2} \leq 10^{-5}$.

References

(1) Oh, S. H.; Lee, K.-C.; Chun, J.; Kim, M.; Lee, S. S. Micro Heat Flux Sensor Using Copper Electroplating in SU-8 Microstructures. *Journal of Micromechanics and Microengineering* **2001**, *11*, 221.

(2) Nagai, H.; Rossignol, F.; Nakata, Y.; Tsurue, T.; Suzuki, M.; Okutani, T. Thermal Conductivity Measurement of Liquid Materials by a Hot-Disk Method in Short-Duration Microgravity Environments. *Materials Science and Engineering: A* **2000**, *276*, 117–123.

(3) Rayleigh, L. LIX. On Convection Currents in a Horizontal Layer of Fluid, when the Higher Temperature is on the under Side. *The London, Edinburgh, and Dublin Philosophical Magazine and Journal of Science* **1916**, *32*, 529–546.

(4) Kleiber, M.; Joh, R. *VDI-Wärmeatlas*, 10th ed.; Springer Berlin Heidelberg: Berlin, Heidelberg, 2006; pp 249–294.

(5) Wen, B.; Hesse, M. A. Rayleigh Fractionation in High-Rayleigh-Number Solutal Convection in Porous Media. 2018; arXiv:1801.03075 [physics.flu-dyn], arXiv, https://arxiv.org/abs/1801.03075, (accessed 14. November 2019).

(6) Rudolph, N.; Osswald, T. A. *Polymer Rheology: Fundamentals and Applications*, 1st ed.; Carl Hanser Verlag GmbH Co KG: Munich, Germany, 2014; pp 61–64.

(7) CLEARCO Viscousity Temperature Chart. `http://www.clearcoproducts.com/pdf/bath-fluids/silicone_fluids_viscosity_temp_chart.pdf`, (accessed 22 March 2020).

(8) Bruus, H. *Theoretical Microfluidics*, 1st ed.; Oxford university press Oxford: New York, USA, 2008; Vol. 18; pp 48–51.

(9) Slattery, J. C. *Advanced Transport Phenomena*, 1st ed.; Cambridge University Press: New York, USA, 1999.

(10) Butt, H.-J.; Graf, K.; Kappl, M. *Physics and Chemistry of Interfaces*, 3rd ed.; John Wiley & Sons: Weinheim, Germany, 2013; p 11.

(11) Casey, J. *Exploring Curvature*, 1st ed.; Vieweg Verlag: Braunscheig, Germany, 1996; p 218.

(12) Hirsch, C. *Numerical Computation of Internal and External Flows: The Fundamentals of Computational Fluid Dynamics*, 2nd ed.; Elsevier: Burlington, USA, 2007; Vol. 1; pp 141–197.

(13) Kondic, L. Instabilities in Gravity Driven Flow of Thin Fluid Films. *SIAM Review* **2003**, *45*, 95–115.

(14) Shampine, L. F.; Reichelt, M. W. The Matlab ODE Suite. *SIAM J. Sci. Comput.* **1997**, *18*, 1–22.
